# Pyrethroid Resistance in Malaysian Populations of Dengue Vector *Aedes aegypti* Is Mediated by CYP9 Family of Cytochrome P450 Genes

**DOI:** 10.1371/journal.pntd.0005302

**Published:** 2017-01-23

**Authors:** Intan H. Ishak, Basile Kamgang, Sulaiman S. Ibrahim, Jacob M. Riveron, Helen Irving, Charles S. Wondji

**Affiliations:** 1 Department of Vector Biology, Liverpool School of Tropical Medicine, Pembroke Place, Liverpool L3 5QA, United Kingdom; 2 School of Biological Sciences, Universiti Sains Malaysia, Penang, Malaysia; 3 Research Unit of Liverpool School of Tropical Medicine at Organisation de Coordination pour la lutte contre les Endémies en Afrique Centrale, Yaoundé, Cameroon; 4 Department of Biochemistry, Bayero University, PMB, Kano, Nigeria; North Carolina State University, UNITED STATES

## Abstract

**Background:**

Dengue control and prevention rely heavily on insecticide-based interventions. However, insecticide resistance in the dengue vector *Aedes aegypti*, threatens the continued effectiveness of these tools. The molecular basis of the resistance remains uncharacterised in many endemic countries including Malaysia, preventing the design of evidence-based resistance management. Here, we investigated the underlying molecular basis of multiple insecticide resistance in *Ae*. *aegypti* populations across Malaysia detecting the major genes driving the metabolic resistance.

**Methodology/Principal Findings:**

Genome-wide microarray-based transcription analysis was carried out to detect the genes associated with metabolic resistance in these populations. Comparisons of the susceptible New Orleans strain to three non-exposed multiple insecticide resistant field strains; Penang, Kuala Lumpur and Kota Bharu detected 2605, 1480 and 425 differentially expressed transcripts respectively (fold-change>2 and p-value ≤ 0.05). 204 genes were commonly over-expressed with monooxygenase P450 genes (CYP9J27, CYP6CB1, CYP9J26 and CYP9M4) consistently the most up-regulated detoxification genes in all populations, indicating that they possibly play an important role in the resistance. In addition, glutathione S-transferases, carboxylesterases and other gene families commonly associated with insecticide resistance were also over-expressed. Gene Ontology (GO) enrichment analysis indicated an over-representation of GO terms linked to resistance such as monooxygenases, carboxylesterases, glutathione S-transferases and heme-binding. Polymorphism analysis of CYP9J27 sequences revealed a high level of polymorphism (except in Joho Bharu), suggesting a limited directional selection on this gene. *In silico* analysis of *CYP9J27* activity through modelling and docking simulations suggested that this gene is involved in the multiple resistance in Malaysian populations as it is predicted to metabolise pyrethroids, DDT and bendiocarb.

**Conclusion/significance:**

The predominant over-expression of cytochrome P450s suggests that synergist-based (PBO) control tools could be utilised to improve control of this major dengue vector across Malaysia.

## Author Summary

*Aedes aegypti* is the major vector of emerging or re-emerging arboviruses such as dengue, chikungunya and Zika viruses worldwide. As there is still no vaccine or specific treatment for these diseases, their prevention relies mainly on insecticide-based interventions. Unfortunately, vector control programs are facing operational challenges with mosquitoes becoming resistant to commonly used insecticides in several regions across the world. To facilitate the design of suitable resistance management strategies for control of this vector, we investigated the molecular basis of insecticide resistance across four Malaysian *Ae*. *aegypti* populations. Transcriptional analysis revealed that 204 genes were commonly over-expressed with cytochrome P450 genes (CYP9J27, CYP6CB1, CYP9J26 and CYP9M4) consistently found as the most up-regulated detoxification genes in all populations indicating that they likely play an important role in the observed resistance. Quantitative real-time polymerase chain reaction (qRT-PCR) validated the over-expression of all cytochrome P450s except for CYP6CB1. Polymorphism analysis of CYP9J27 sequences revealed a high polymorphism (except in Joho Bharu), suggesting a lack of directional selection on this gene. The predominant expression of cytochrome P450s suggests that synergist-based control tools could be used to improve control of this major dengue vector across Malaysia.

## Introduction

The mosquito *Aedes aegypti* Linnaeus, 1762 (Diptera: Culicidae) is the most important vector of dengue, Zika, yellow fever [1, 2] and chikungunya [3, 4] viruses to human throughout the tropical world. This domestic mosquito usually bites during daylight, feeding predominantly on humans, mating and resting indoors and breeding in man-made containers in and around human dwellings [5].

Control of *Ae*. *aegypti* relies on reducing breeding sites and insecticide-based interventions such as treatment of breeding sites, Ultra Low Volume (ULV) space sprays, fogging and thermal spraying [6]. Unfortunately, many vector control programs are threatened by the development of insecticide resistance in *Ae*. *aegypti* [7, 8]. Resistance to multiple insecticides such as pyrethroids, dichlorodiphenyltrichloroethane (DDT), bendiocarb and organophosphates has been reported in *Ae*. *aegypti* [9–12]. Resistance to pyrethroid insecticides, the primary insecticide class used against adult mosquitoes is particularly worrying in the context of the re-emergence of dengue and other arboviruses worldwide, including Zika virus [13, 14]. The elucidation of the mechanisms of insecticide resistance may aid in the in the design of suitable resistance management strategies to prolong the effectiveness of the existing insecticide-based control tools.

The two main causes of insecticide resistance are alterations in the target sites and increase in the rate of insecticide metabolism [15]. Target site resistance is caused by mutations in target genes such as the voltage gated sodium channel (VGSC) which causes knockdown (*kdr*) resistance, mutations in the acetylcholinesterase (*Ace-1*) gene and GABA receptors [15, 16]. The most important target site resistance for mosquitoes is *kdr* as it confers resistance to both pyrethroids and DDT. *kdr* occurs as a result of a change in the affinity of the insecticides to their binding sites, because of mutations in the sodium channel [17]. Several *kdr* mutations have been identified in *Ae*. *aegypti*, and the association between the V1016G/I and the F1534C mutations and pyrethroid resistance has been established [18–20]. In Malaysia, a recent study revealed that the frequency of the 1534C resistant mutation ranges from 40 to 80% whereas the 1016G mutation is found at around 20 to 39% [10]. A significant correlation was also established between F1534C genotypes and pyrethroid resistance [10] in Malaysian mosquitoes, whereas no significant correlation was found for the V1016G mutation. However, an additive effect to pyrethroid resistance was observed when both 1534C and 1016G were present [10].

Another key resistance mechanism is metabolic resistance through up-regulation of detoxification genes. The three main enzymes families responsible for insecticide resistance in mosquitoes are the monooxygenases (cytochrome P450s), glutathione S-transferases (GSTs) and carboxylesterases (COEs) [21, 22]. Metabolic resistance can occur as a result of point-mutations affecting protein activity (e.g. change in binding affinity or an altered substrate specificity) [23–25] or via mutations in *cis/trans* regulatory loci of these three enzyme families [15]. In the case of cytochrome P450s elevated expression of several genes from this family has previously been shown to be primarily responsible for resistance towards pyrethroids, carbamates and organophosphates [7, 15, 26]. Multiple and widespread resistance to insecticides was recently reported in *Ae*. *aegypti* populations across Malaysia [10]. Although the F1534C *kdr* mutation was shown to play some role in the case of pyrethroids and DDT resistance, PBO synergist assays suggested that metabolic resistance mechanisms play an important role in the resistance patterns. Furthermore, it remains unknown whether differences observed in resistance profiles notably in Kuala Lumpur (KL), where high and multiple resistance was observed, is supported by differences in underlying molecular basis of the resistance.

Therefore, to aid the design of suitable resistance management strategies for control of *Ae*. *aegypti* in Malaysia, we investigated the molecular basis of multiple insecticide resistance using a microarray-based genome-wide transcriptional analysis of various populations of this species across Malaysia. This study detected key resistance genes and revealed that metabolic resistance is primarily driven by the cytochrome P450 gene family.

## Materials and Methods

### Ethical clearance

An institutional clearance for this study, including the sampling of mosquito, was granted by the Ministry of Health, Malaysia.

### Mosquito samples

**Fig 1:**
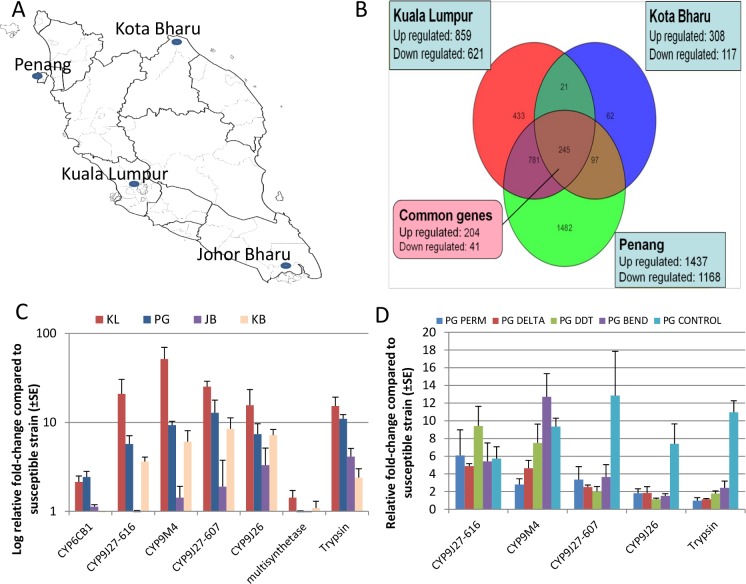
Transcription profiling of *Ae*. *aegypti* in Malaysia. **A) Map of the Malaysian peninsular showing the four collection sites of Penang (PG), Kota Bharu (KB), Kuala Lumpur (KL) and Johor Bharu (JB).** B) Venn diagram of differentially transcribed genes from microarray data (p = 0.01); C) Relative fold-change of candidate genes from qRT-PCR analysis; D) Relative fold-change of candidate genes from qRT-PCR analysis for resistant samples from Penang. Perm stands for Permethrin, Delta for deltamethrin, Bend for bendiocarb.

*Ae*. *aegypti* mosquitoes were collected using ovitraps in July and August 2010 in four states (Fig 1A), namely: Penang (Northwest), Kota Bharu (Northeast), Kuala Lumpur (Central) and Johor Bharu (South) in Malaysia as previously described [10]. Old tyres, flower pots, tree holes and containers that held water were also inspected for larvae. The larvae were reared at the Vector Control Research Unit (VCRU), Universiti Sains Malaysia, in Penang (Malaysia) as recently described [10]. Adult *Ae*. *aegypti* were induced to lay eggs on filter papers that were later dried and shipped to the Liverpool School of Tropical Medicine (LSTM) under the LSTM import license from DEFRA. The egg batches were then allowed to hatch in the insectary in water supplemented with hay infusion solution. Larvae were reared as above and the adults fed with 10% sucrose, and kept at a room temperature of 27 ± 2°C with relative humidity of 70 ± 10%. The bioassays were performed in the same conditions with F_1_ generation or subsequent F_2_ generations. Analysis revealed that all four populations were resistant to permethrin and to deltamethrin. The highest resistance levels to both insecticides were observed in Kuala Lumpur with nearly all mosquitoes surviving the 1 h exposure. However, in Kota Bharu, the high permethrin resistance (10% mortality) contrasted with only a moderate resistance to deltamethrin (82% mortality). These populations were also resistant to DDT with the highest resistance level recorded again in Kuala Lumpur with no mortality after 1 h exposure. Widespread resistance is also observed against the carbamate bendiocarb except in Kota Bharu where 91% mortality was observed in females. Full susceptibility was observed for the organophosphate malathion, except for Kuala Lumpur where a probable resistance is observed with 91% mortality. Similarly, a full susceptibility was observed against dieldrin except in Johru Bharu where a moderate resistance is observed with 88% mortality in females [10]. These samples have been used for the transcriptional analysis.

### Investigating metabolic resistance using genome-wide transcriptional analysis

A series of microarray experiments were conducted to identify the genes potentially associated with the metabolic resistance in the *Ae*. *aegypti* populations across Malaysia. The genome-wide transcription profiling was carried out in comparison with a susceptible strain (New Orleans) to investigate the differential expression profiles of the populations. Microarray hybridizations were done using the 8 x 15k Agilent *Ae*. *aegypti* chip containing eight replicated arrays of 60-mers oligo-probes representing more than 14,320 different *Ae*. *aegypti* transcripts from AaegL1.2 Vectorbase annotation and several control (ArrayExpress accession number A-MEXP-1966). This 8 x 15k microarray enables a high coverage across the whole genome [27] and at the same time reduces cost. It also increases throughput compared to the previous *Aedes* Detox chip which includes only 204 detoxification genes probes [26].Total RNA was extracted from 3 replicates of pools of 10 adult unexposed mosquitoes from all four strains. Only samples from Penang (PG), Kuala Lumpur (KL) and Kota Bharu (KB) were used in the microarray. Samples from Johor Bharu (JB) were omitted from the microarray experiment due to the population having similar resistance profile to PG (but at a lower level) and therefore possibly sharing similar resistance mechanisms [10]. However, samples from JB were used in the validation of the candidate genes through qRT-PCR. RNA from the susceptible New Orleans (NO) strain was also used. Quality and quantity of RNA were assessed using the Nanodrop ND-1000 (Thermo Scientific, Delaware, USA) and the Agilent 2100 Bioanalyzer (Agilent Technologies, California, USA). RNA (100ng) sample from each sample were amplified and labelled using the two-colour low input Quick Amp Labelling kit (Agilent Technologies). Labelled cRNAs were hybridized to the arrays for 17h at 65°C according to the manufacturer’s protocol. Three different comparisons were made: NO susceptible lab strain vs. PG, NO vs. KL and NO vs. KB (non-exposed vs susceptible) (S1 Fig). A total of five replicates (including three biological replicates and two dye swaps) were performed for each comparison as previously successfully done in other species to detect the resistance genes [28, 29].

### Microarray data analysis and gene ontology (GO) enrichment analysis

Microarray data were analysed using the Genespring GX 12.0 software (Agilent Technologies, US). Mean expression ratios were assessed using a t-test against zero with a multiple testing correction (Benjamini-Hochberg false discovery rate). Genes showing both t-test p-values less than 0.05 and a fold change (FC) value greater than 2 were considered significantly differentially transcribed between the two samples compared.

Genes or entities that were considered as significantly differentially expressed were used for Gene Ontology (GO) enrichment analysis Blast2GO software (BioBam Bioinformatics S.L., Valencia, Spain). Descriptions and GO-terms of transcript-IDs were generated from Blast2GO extracted from VectorBase. GO term enrichment analysis was performed on the significantly up-regulated genes (72% of transcripts present on microarray have GO-terms) using Blast2GO software with Fisher’s exact test and false discovery rate (FDR) < 0.05 [30].

### Validation of candidate genes using quantitative real time-polymerase chain reaction (qRT-PCR)

Some of the most significantly differentially expressed genes from the microarray analysis were selected for qRT-PCR validation. Firstly, control samples not exposed to insecticides from all four locations (including the JB sample that was not used in the microarray to also confirm their potential role in this location) were compared to the susceptible NO strain to assess the geographical variation of these key genes. Secondly, samples alive after exposure to various insecticides were also compared to the control non exposed mosquitoes and to the NO strain to detect possible induction of these genes or whether their over-expression was more associated to a specific insecticide than others. This was done only in PG as this population is moderately resistant to all insecticides allowing a comparison to the control sample. Total RNA from 3 replicates of each samples were used for the qRT-PCR. Primers used are listed in S1 Table. Standard curves for each gene were generated using a serial dilution of cDNA to assess PCR efficiency and quantitative differences between samples. qRT-PCR amplification was performed as described previously [28, 29]. The relative expression level and fold change (FC) of each target gene in field samples relative to the susceptible NO (S) were calculated according to the 2^-^ΔΔ^CT^ method incorporating the PCR efficiency [31] after normalization with the housekeeping genes ribosomal protein S7 (*RSP7*; AAEL009496-RA) and Tubulin (AAEL009496-RA).

### Polymorphism analysis of the candidate resistance P450 gene *CYP9J27*

Patterns of polymorphism for CYP9J27 were explored across Malaysian *Ae*. *aegypti* populations to detect possible correlation with resistance profile using the permethrin susceptible (NO) and unexposed *Ae*. *aegypti* mosquitoes from the four sites in Malaysia: JB, KL, KB and PG. Full-length coding region of *CYP9J27* was amplified from cDNA using the same cDNA synthesized for qRT-PCR with the Phusion High-Fidelity DNA Polymerase (Thermo Scientific), cloned into the pJET1.2/blunt cloning vector (Thermo Scientific), and sequenced as described previously [24]. Primers used are listed in S2 Table. Polymorphic positions were detected through manual analysis of sequence traces using BioEdit version 7.2.1 and as sequence differences in multiple alignments using ClustalW [32]. Basic sequence statistics, including the number of haplotypes (h), the number of polymorphism sites (S), haplotype diversity (Hd) and nucleotide diversity (π), were computed with DnaSP 5.10 [33]. The statistical tests of Tajima [34], Fu and Li [35] was used with DnaSP to test non-neutral evolution and deviation from mutation-drift equilibrium. Different haplotypes were compared by constructing a maximum likelihood phylogenetic tree and polymorphic positions of amino acid sequences were generated using MEGA 6.0 [36].

### Homology modelling of *CYP9J27* and docking simulations with various insecticides

To predict the ability of the *CYP9J27* to bind the various insecticides homology models of the P450 were created using query amino acid sequences from the various study locations (KL, PG, JB, KB), as well as the sequence from susceptible strain, NO. The 3D models of the P450s were created using the standalone tool EasyModeller [37]. CYP3A4 (PDB: 1TQN) [38] was used as a template with sequence identity of 31% for all the five CYP9J27 amino acid sequences. Virtual datasets of ligand insecticides: 1*R*-*cis* permethrin (ZINC01850374), deltamethrin (ZINC01997854), DDT (ZINC01530011) and bendiocarb (ZINC02015426) were retrieved from the library in ZINC^12^ database (https://zinc.docking.org/) [39]. Docking simulations were carried out using the Blind Docking Web Server (http://bio-hpc.ucam.edu/webBD/index.php/entry). For each ligand, 30 binding poses were generated and sorted according to the binding energy and conformation in the protein’s active site. Figures were prepared using the PyMOL 1.7 [40].

## Results

### 1-Genome-wide microarray-based transcriptional profiling

The microarrays were used to perform a genome-wide transcription analysis between the susceptible strain (New Orleans, NO) and non-exposed field populations from Penang (PG), Kuala Lumpur (KL) and Kota Bharu (KB). The number of differentially transcribed genes after analysis is presented in the Venn diagram (Fig 1B). Penang had the most number of differentially transcribed probes with 2605 probes, followed by Kuala Lumpur with 1480 gene probes, and Kota Bharu with 425 gene probes. The number of commonly up-regulated probes in all population is 204 while 41 probes were down-regulated.

#### Genes commonly up-regulated in the three locations

To detect the genes associated with insecticide resistance across Malaysian *Ae*. *aegypti*, priority was given to the genes commonly up-regulated in the three locations since their consistent over-expression increases the likelihood of them playing a role in the observed resistance. The 204 up-regulated gene probes comprise various gene families such as those involved in protein synthesis, ion transport, detoxification, etc (S3 Table). The most commonly over-expressed gene was the anionic-trypsin with very high fold change notably in KL (FC = 759) and PG (FC = 581) but with moderate over-expression in KB populations (FC = 43.8). This gene is known to be expressed abundantly in mosquito’s midgut where it hydrolyses proteins after blood meals [41]. Over-expression of midgut proteases has been commonly associated with insecticide resistance in various mosquito species [28, 29, 41, 42]. The list of the top 50 up-regulated probes include several probes associated with regulation of gene expression including the transcription elongation regulator 1 (FC = 448 in KL), five finger zinc proteins and two probes from the GATA transcription factor. It is possible that these genes are involved in the transcriptional regulation of the major insecticide detoxification genes in these populations of *Ae*. *aegypti*. Among the commonly up-regulated detoxification genes, cytochrome P450s were the most predominant with nine genes over-expressed, while only a single carboxylesterase was over-expressed. Of the P450s, *CYP6CB1* was the most over-expressed gene with the highest FC value in PG (212.00) followed by Kuala Lumpur and Kota Bharu with FC values of 124.70 and 36.40, respectively. Another detoxification gene that was over-expressed was the *CYP9J26* gene (AAEL014609-RA) with a similar expression level in PG and KL with 7.20 and 7.70 FC values. The expression was lower for this gene in KB with FC value of 4.40. *CYP9M4* and *CYP9J27-616* had the similar expression level in the three locations. In KL the FC value for *CYP9M4* and *CYP9J27-616* (AAEL014616) was 13.20 and 13.80, followed by PG with 7.20 and 7.40 and KB with 4.10 and 3.70. Another cytochrome P450 which was over-expressed in all the locations was the transcript AAEL014614-RA which had the closest hit to *CYP9J4* in *An*. *gambiae* after NCBI BLAST. The FC value for this gene was highest in KL with 13.80 followed by PG and KB with 2.90 and 2.10 FC values respectively. The unique carboxylesterase gene (AAEL004724-RA) commonly up-regulated in the three locations had similar low FC values for all the locations ranging from 2.00 to 2.80 (Table 1).

**Table 1:** Probes from detoxification genes & genes linked with resistance up-regulated in Malaysian populations in comparison with susceptible New Orleans strain. FC = fold change (p = 0.01).

Probe Name	Systematic Name	Blast2GO Annotation	Kota Bharu vs NO		Kuala Lumpur vs NO		Penang vs NO	
			Absolute FC	Corrected p-value	Absolute FC	Corrected p-value	Absolute FC	Corrected p-value
**Common to all three locations**								
CUST_2845_PI424980000	AAEL009018-RA	cytochrome p450 (CYP6CB1)	36.40	0.04881	124.70	0.00128	212.00	0.00076
CUST_9292_PI424980000	AAEL003349-RA	nadph-cytochrome p450 reductase	6.60	0.0442	24.20	0.00421	8.90	0.00012
CUST_12457_PI424980000	AAEL014689-RA	nadph cytochrome p450	5.30	0.0303	15.10	0.00163	11.00	0.00022
CUST_105_PI424980000	AAEL014609-RA	cytochrome p450 (CYP9J26)	4.40	0.01914	7.70	0.00435	7.20	0.00033
CUST_157_PI424980000	AAEL001320-RA	cytochrome p450 (CYP9M4)	4.10	0.01679	13.20	0.00317	7.20	0.00174
CUST_106_PI424980000	AAEL014616-RA	cytochrome p450 (CYP9J27)	3.70	0.01236	13.80	0.00178	7.80	0.00022
CUST_140_PI424980000	AAEL014614-RA	cytochrome p450(as CYP9J4 in *An*. *gambiae*)	2.10	0.00352	13.80	0.00178	2.90	0.00104
CUST_228_PI424980000	AAEL004724-RA	carboxylesterase	2.00	0.00243	2.10	0.00808	2.80	0.00046
**Common to KB and KL but not PG**								
CUST_151_PI424980000	AAEL001288-RA	cytochrome p450(as CYP9M1 in *An*. *gambiae*)	2.50	0.00908	2.40	0.00835		
**Common to KB and PG but not KL**								
CUST_10688_PI424980000	AAEL012836-RA	cytochrome b561	4.40	0.00986			9.50	0.00022
CUST_67_PI424980000	AAEL014893-RA	cytochrome p450 (CYP6BB2)	2.00	0.00104			5.20	0.00019
**Common to KL and PG but not KB**								
CUST_162_PI424980000	AAEL014607-RA	cytochrome p450 (CYP9J27)			356.70	0.00214	395.70	0.00022
CUST_145_PI424980000	AAEL014606-RA	cytochrome p450 (CYP9J7)			16.90	0.00349	12.20	0.00069
CUST_131_PI424980000	AAEL008023-RA	cytochrome p450 (CYP4C52)			12.70	0.00512	3.50	0.00376
CUST_148_PI424980000	AAEL014891-RA	cytochrome p450 (as CYP6P4 in *An*. *gambiae*)			8.50	0.00978	9.70	0.00117
CUST_64_PI424980000	AAEL007473-RA	cytochrome p450 (CYP6AH1)			5.90	0.00752	2.50	0.0024
CUST_176_PI424980000	AAEL007951-RA	glutathione-s-transferase gst (GSTE2)			4.50	0.00397	2.40	0.00291
CUST_256_PI424980000	AAEL004118-RA	aldo-keto reductase			3.20	0.00252	5.00	0.0006
CUST_352_PI424980000	AAEL008672-RA	abc transporter			2.50	0.00711	3.40	0.00056
**KB only**								
CUST_8318_PI424980000	AAEL014246-RA	glucosyl glucuronosyl transferases	3.60	0.04007				
CUST_348_PI424980000	AAEL004331-RA	abc transporter	3.20	0.00636				
CUST_112_PI424980000	AAEL001292-RA	cytochrome p450 (CYP9M7)	3.00	0.03684				
**KL only**								
CUST_143_PI424980000	AAEL014617-RA	cytochrome p450 (CYP9J28)			10.80	0.00209		
CUST_6_PI424980000	AAEL004870-RA	cytochrome p450 (CYP18A1)			3.20	0.00725		
CUST_7415_PI424980000	AAEL010157-RA	microsomal glutathione s-transferase			2.80	0.00212		
CUST_111_PI424980000	AAEL001312-RA	cytochrome p450 (CYP9M6)			2.40	0.00402		
**PG only**								
CUST_88_PI424980000	AAEL012491-RA	cytochrome p450 (CYP6P12)					6.50	0.00091
CUST_67_PI424980000	AAEL014893-RA	cytochrome p450 (CYP6BB2)					5.20	0.00019
CUST_86_PI424980000	AAEL009126-RA	cytochrome p450 (CYP6N6)					4.90	0.00041
CUST_129_PI424980000	AAEL005695-RA	cytochrome p450 (CYP325X1)					4.60	0.00542
CUST_184_PI424980000	AAEL011741-RB	glutathione s-transferase (GSTS1)					4.30	0.00456
CUST_233_PI424980000	AAEL000905-RA	carboxylesterase					4.00	0.00082
CUST_3471_PI424980000	AAEL005937-RA	atp-binding cassette transporter					3.60	0.00039
CUST_44_PI424980000	AAEL007795-RA	cytochrome p450 (CYP4D37)					3.40	0.00936
CUST_338_PI424980000	AAEL008624-RA	abc transporter					3.40	0.00029
CUST_87_PI424980000	AAEL009121-RA	cytochrome p450 (CYP6N9)					3.50	0.00045
CUST_44_PI424980000	AAEL007795-RA	cytochrome p450 (CYP4D37)					3.40	0.00936
CUST_31_PI424980000	AAEL017136-RA	cytochrome p450 (CYP325V1)					3.10	0.00046
CUST_95_PI424980000	AAEL006798-RA	cytochrome p450 (CYP9J10)					3.00	0.00064
CUST_11833_PI424980000	AAEL014612-RA	cytochrome p450 (as CYP9J5 in *An*. *gambiae*)					2.90	0.00109
CUST_93_PI424980000	AAEL009131-RA	cytochrome p450 (CYP6Z8)					2.50	0.00639
CUST_6046_PI424980000	AAEL009119-RA	cytochrome p450 (as CYP6M2 in *An*. *gambiae*)					2.40	0.00168
CUST_10102_PI424980000	AAEL012838-RA	cytochrome b561 as CYP325K1 in *An*. *gambiae*)					2.30	0.00055
CUST_130_PI424980000	AAEL008017-RA	cytochrome p450 (CYP4C50)					2.30	0.00943
CUST_102_PI424980000	AAEL017217-RA	cytochrome p450 (as CYP9J5 in *An*. *gambiae*)					2.30	0.00039
CUST_7589_PI424980000	AAEL015641-RA	cytochrome p450 (as CYP6AH1 in *An*. *gambiae*)					2.20	0.00047
CUST_108_PI424980000	AAEL002638-RA	cytochrome p450 (CYP9J6)					2.20	0.00046
CUST_4_PI424980000	AAEL002031-RA	cytochrome p450 (CYP12F7)					2.10	0.00123
CUST_346_PI424980000	AAEL006717-RA	abc transporter					2.10	0.00138
CUST_194_PI424980000	AAEL002367-RA	Carboxylesterase (CCEAE1B)					2.10	0.00228
CUST_357_PI424980000	AAEL005249-RA	abc transporter					2.00	0.00778

### Genes commonly up-regulated only in two locations

#### Commonly up-regulated only in KL and PG but not KB

From the list of probes over-expressed only between KL and PG but not in KB, eight genes linked to detoxification and resistance were observed. Another transcript of *CYP9J27-607* (AAEL014607-RA) was the most over-expressed cytochrome P450 with FC values more than 350 in both locations which is by far more than the FC values for the same gene but with a different transcript (AAEL014616-RA) in the commonly over-expressed genes for all three locations. Similarly, a different transcript of the *CYP9J7* (AAEL014606-RA) is also up-regulated in KL (FC value 16.90) and PG (FC value 12.20) only. Other cytochrome P450s included *CYP4C52*, *CYP6AH1* and a transcript with the closest hit to *CYP6P4* in *An*. *gambiae*, a cytochrome P450 associated with permethrin resistance in *An*. *gambiae* [43] and recently showed to metabolise pyrethroids in *An*. *arabiensis* [25, 44]. Also, the glutathione S-transferase gene *GSTe2*, a known DDT metaboliser [45] [23] was commonly up-regulated in KL and PG, however with low FC values of 4.50 and 2.40 respectively (Table 1).

#### Genes commonly up-regulated only in KB and PG but not KL

Only two genes were established as commonly over-expressed in KB and PG. One cytochrome P450 *CYP6BB2* with FC value of 2.00 in KB and 5.20 in PG was recorded. Another gene up-regulated was the cytochrome b561 which has been previously established to play a role in insecticide resistance in *Ae*. *aegypti* [23, 30] (Table 1).

#### Commonly up-regulated only in KL and KB but not PG

Only one cytochrome P450 transcript with the closest hit to *CYP9M1* in *An*. *gambiae* was commonly up-regulated in KL and KB. The FC values were low with 2.50 in KB and 2.40 in KL (Table 1).

### Genes up-regulated only in a single location

From the list of the genes up-regulated in a single location only, PG had the most number of up-regulated detoxification genes exclusively expressed in this location with a predominance of cytochrome P450 among which is the P450 *CYP6P12* (FC value 6.50). The ortholog of this gene in *Ae*. *albopictus* was recently shown to be the main pyrethroid resistance gene in this species in KL [42]. *CYP6P12* is also the ortholog of *CYP6P4* in the malaria vectors *An*. *gambiae*, *An*. *arabiensis* and *An*. *funestus*. Other cytochrome P450 genes are the *CYP6BB2* (a different probe than in the commonly up-regulated in PG and KB), *CYP6N6*, *CYP325X1*, *CYP4D37*, *CYP9J10*, *CYP6Z8* and others. Other genes belong to GSTs (*GSTS1*), carboxylesterase (transcript AAEL000905-RA and CCEAE1B) and the ABC transporters (Table 1).

The set of probes up-regulated only in KL is mainly made of cytochrome P450s among which the *CYP9J28* gene exhibited the highest FC value of 10.80. This gene has previously been shown to confer pyrethroid resistance in *Ae*. *aegypti* [46]. Other cytochrome P450s over-expressed only in KL are *CYP18A1* and *CYP9M6* (Table 1).

The genes up-regulated in KB only are a cytochrome P450 (*CYP9M7*), an ABC transporter and a UDP glucuronosyl transferase (Table 1).

#### Genes commonly down-regulated in the three locations

Among the top genes commonly down-regulated in PG, KL and KB the highest down-regulated was the domain-containing protein cg6693. Glutamine synthetase seems to be consistently down-regulated with two probes being consistently at the top. One detoxification gene *CYP325M2* was found to be the commonly down-regulated in all three locations (S4 Table and S5 Table). Among the detoxification genes down-regulated only in one or two locations, were several cytochrome P450s notably from CYP325 (CYP325F2, CYP325G2, CYP325C2, CYP325T2 and more), CYP4J (CYP4J10, CYP4J13, CYP4J14, CYP4J15) and some CYP6 (CYP6N13, CYP6N14, CYP6N17, CYP6AG8, CYP6BY1).

### 2-Validation of candidate genes through qRT-PCR

Seven candidate genes commonly up-regulated in all three locations were chosen to validate the microarray expression pattern. These genes were trypsin (AAEL013623-RA), multisynthetase complex, *CYP6CB1*, *CYP9J26* (AAEL014609-RA), *CYP9J27-607* (AAEL014607-RA), *CYP9M4* and *CYP9J27-616 (AAEL014616-RA)*. The most over-expressed P450 for microarray in all 3 locations, *CYP6CB1* was not significantly over-expressed from the qRT-PCR results in all four locations including JB (Fig 1C). However, the over-expression of other five P450 genes was confirmed whereas the over-expression of the multisynthetase gene was not confirmed. The JB sample consistently exhibited a lower expression level for all these genes in comparison to the other 3 locations. This difference could be associated with the lower resistance level to pyrethroids and DDT in JB. Overall, for most of the genes, the highest over-expression was observed for the KL population which correlates with the high resistance level to pyrethroids and DDT observed in KL from bioassays [10]. The expression profile of the five significantly over-expressed genes from qRT-PCR was further assessed for various samples from mosquitoes alive after exposure to different insecticides. From this analysis, *CYP9J27-616* was more over-expressed, although not significantly, in the DDT resistant mosquitoes from PG than the other insecticides (Fig 1D). *CYP9M4* was significantly more over-expressed in the PG bendiocarb resistant sample than other mosquito samples. No significant difference was observed between samples for *CYP9J26*, *CYP9J27-607* and for the trypsin gene.

### 3. Gene ontology (GO) enrichment analysis

GO enrichment analysis was used to identify particular Gene Ontology (GO) terms that were over represented in the data set of transcripts up-regulated in all three resistant populations and in single populations. When comparing the commonly up-regulated genes in all three populations at p = 0.01, a few GO terms that relates to detoxification was observed. These included NADPH-hemoprotein reductase activity, ATP binding and others (S2 Fig). When observing the GO terms of single locations either in PG (S3 Fig), KB (S4 Fig) or KL (S5 Fig), interesting terms such as ATP binding, heme-binding and monooxygenase activity were over-represented possibly associated with the multiple resistance to insecticides detected in these locations.

### 4-Genetic variability of *CYP9J27* in relation to pyrethroid resistance

**Fig 2:**
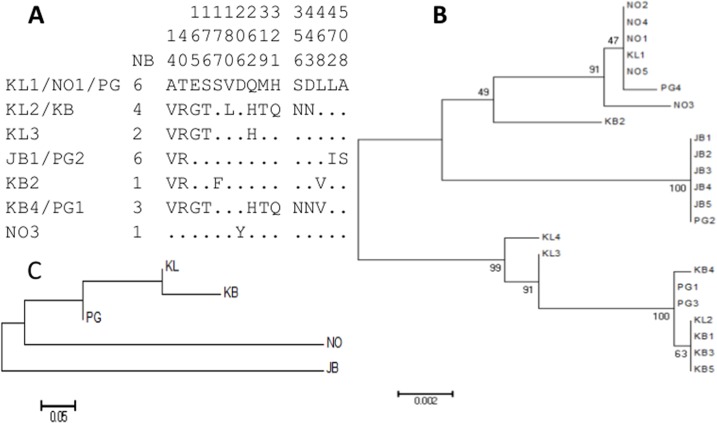
Genetic diversity of *CYP9J27* across Malaysian populations of *Aedes aegypti*. A) Schematic representation of *CYP9J27* haplotypes from across Malaysia and in the NO susceptible strain. The polymorphic positions for CYP9J27 amino acid sequences are highlighted. A number is given to each haplotype preceded by location origin (KL, KB, JB, PG and NO, for Kuala Lumpur, Kota Bharu, Johor Bharu, Penang and New Orleans, respectively). The column Nb stands for the number of individual mosquitoes sharing a haplotype. B) Neighbor joining tree of *CYP9J27* haplotypes. C) Genetic distance tree between the different samples based on the *Nst* genetic distances.

In order to functionally investigate the potential involvement of the commonly over-expressed CYP9J27 gene in conferring resistance to insecticides in *Ae*. *aegypti*, further attention was paid on this gene since particularly as the role of other P450s has already been assessed [46]. Full-length cDNA sequences (1611 bp) of *CYP9J27-616* (hereafter CYP9J27), one of the genes commonly over-expressed all the three populations from microarray were successfully generated for 23 clones from four locations across Malaysia and the susceptible NO strain allowing to assess their polymorphism (GenBank Accesion Number: KX394421-KX394443). Overall, analysis of the genetic variability revealed 54 polymorphic sites (s), 10 haplotypes (h), and 15 amino acid changes in total, with the lowest polymorphism observed in JB (Table 2, Fig 2A). Phylogenetic tree constructed using maximum likelihood confirm the relatively high genetic variability of this gene in each location except in JB (Fig 2B). Haplotype diversity is high (Hd>0.73) in most samples except JB suggesting that little directional pressure is acting in this population. However, the lack of diversity in JB should be further investigated. The difference between JB and other populations is further highlighted on the neighbour-joining tree of the genetic distances between various populations, with JB exhibiting high level of genetic differentiation based on *K*_*ST*_ estimates whereas KL and KB cluster together. When all the sequences were analysed as a unique sample, the Tajima D, was positive (D = 2.0675) and statistically significant (P<0.05) (Table 2). In the other hand, when the sequences were analysed according to the origin of each sample this statistics was negative but not statistically significant (Table 2). Negative values for these indexes may indicate an excess of rare polymorphisms in a population and suggest either population expansion or background selection [35]. However, further analysis with more sequences is needed to test for a signature of selection on this gene.

**Table 2:** Summary statistics for polymorphism of *CYP9J27* haplotypes from unexposed samples and susceptible NO strains, across Malaysia.

Samples	N	S	h	Hd	Syn	Nonsyn	π (κ)	D	D*
**Johor Bharu**	5	0	1	0.00	0	0	0.0000 (NA)	NA	NA
**Kuala Lumpur**	4	36	4	1.00	26	10	0.1159 (18.66)	-0.5119	0.5119
**Kota Bharu**	5	30	3	0.70	20	10	0.0077 (12.40)	-1.0384	-1.0384
**Penang**	4	45	3	0.83	34	11	0.0163 (26.33)	0.7572	0.7572
**New Orleans**	5	5	2	0.40	4	1	0.0012 (2.00)	-1.1239	-1.1239
**All**	**23**	**54**	**10**	**0.87**	**39**	**15**	**0.0138 (22.33)**	2.0675*	**1.3035**

N = number of sequences (n); S, number of polymorphic sites; h, haplotype; Hd, haplotype diversity; Syn, Synonymous mutations; Nonsyn, Non-synonymous mutations; π, nucleotide diversity (k = mean number of nucleotide differences); Tajima’s D and Fu and Li’s D statistics, ns, not significant.

* Statistically significant at p<0.05; NA, not applicable.

### 5. Prediction of *CYP9J27* activity through modelling and docking simulations with various insecticides

**Fig 3:**
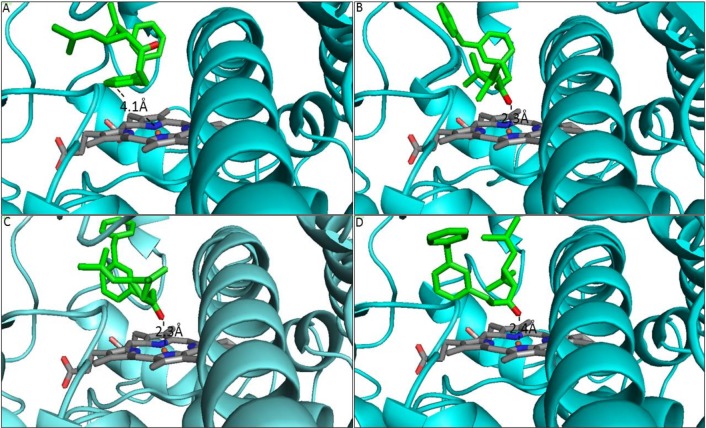
**Binding conformation of permethrin in (A) KL, (B) JB, (C) PG and (D) NO CYP9J27 models.** Distances between the probable sites of attack on permethrin and the heme iron are annotated.

For each of the four locations as well the NO, CYP9J27 model with the highest ERRAT score (which is based on the patterns of non-bonded interaction) [47] was chosen for molecular docking. For the type I pyrethroid permethrin, the KL model exhibited the lowest binding energy (10.1 kcal/mol) which was comparable to values obtained from JB and KB (-9.9 kcal/mol respectively); but lower than observed with PG (-9.6 kcal/mol) (S6 Table). NO model exhibited the highest binding energy of -9.1 kcal/mol, however this binding energy predicts good binding of the permethrin to the model from the susceptible strain, even though lower than obtained from the other models. However, with the exception of KL model in which permethrin binds with the benzyl ring perpendicular above the heme plane (2 spot of benzyl ring located 4.1Å from the heme iron (Fig 3A), predicting ring hydroxylation to generate 2-hydroxypermethrin), the insecticide docked in all the other models with the ester oxygen projecting toward the heme iron, susceptible to ester hydrolysis (Fig 3B–3D; S9A Fig).

As with permethrin, docking of type II pyrethroid deltamethrin to the active site of KL CYP9J27 model produced the lowest binding energy (-10.3 kcal/mol), lower than observed with the other models, with NO model producing the highest binding free energy (-9.3 kcal/mol) (S6 Table). Also, as with permethrin, deltamethrin docked in the active site of KL model with the phenoxy ring above the heme iron and the 4´ spot located within 4.2Å of the heme iron (S6A Fig). In this posture ring hydroxylation to generate 4´-hydroxydeltamethrin is predicted. For JB and the NO models the α-cyano group docked above the heme iron within 2.4Å of the heme iron (S6B and S6D Fig), while for PG it is the ester oxygen which projects toward the heme iron at a distance of 2.3Å (S6C Fig). For KB, it’s the benzyl ring which docked within 2.9Å from the heme iron with possibility of ring hydroxylation (S9B Fig).

With the exception of NO model, DDT docked in the models of KL, JB, PG and KB with low binding energy (S6 Table) and productively with carbon atom of the trichloromethyl group positioned within a distance of 5.4Å, 4.3Å and 4.8Å respectively for KL, JB and PG (S7A–S7C Fig). The same pose was obtained with KB model with the carbon atom of the trichloromethyl group at a distance of 5.6Å from the heme iron (S9C Fig). In this posture reductive dechlorination to generate DDE is predicted. In contrast, DDT docked unproductively in the active site of NO with the chlorine atoms projecting toward the heme iron at a distance of 3.6Å (S7D Fig). Generally, the free energy of DDT binding in the models from the resistant populations is higher than obtained with the pyrethroids but low enough to warrant good binding, with the exception of NO model to which the binding energy of DDT is very high (-5.3 kcal/mol).

In KL, JB, PG and KB models, bendiocarb bind productively with the carbamic acid ester group oriented toward heme at a reasonable distance of 3.4Å, 3.9Å, 3.7Å and 3.9Å, respectively (S8A–S8C and S9D Figs). In this posture ester hydrolysis to generate benzodioxol-4-ol and carbamic acid is predicted. Free binding energy for all the models is lower than obtained from docking with pyrethroids and DDT; however, NO has the highest binding energy of all (-5.1 kcal/mol) indicative of low affinity compared with the other models. Not surprisingly, bendiocarb docked in the active site of NO model away (13.2Å) from heme catalysis centre for any meaningful interaction to take place.

## Discussion

Elucidating the molecular basis of insecticide resistance in the dengue vector *Ae*. *aegypti* is an important step for the design of suitable resistance management strategies to control this vector. In this study genome-wide transcriptional analyses carried out using microarray showed that metabolic resistance plays an important role in conferring resistance to insecticides in *Ae*. *aegypti* across Malaysia. This further supports the synergist assay result with PBO showing a recovery of susceptibility notably for pyrethroids [10]. The role of metabolic resistance was supported by the over-expression of many genes belonging to detoxification families in PG, KL and KB when comparing them to the susceptible NO strain. The most prominent detoxification gene family established in this *Aedes* populations were the cytochrome P450 genes which were the only detoxification family discovered except for one unique carboxylesterase commonly over-expressed in the three locations.

The most over-expressed cytochrome P450 is the *CYP6CB1* which has also been reported in a strain from Isla Mujeres in Mexico [46]. Unfortunately, the microarray over-expression of *CYP6CB1* was not supported by the qRT-PCR validation for all four populations tested. This discrepancy between the two methods could be caused by differences in the sequences of the microarray probes and the qRT-PCR primers. Recent functional analysis has shown that this *CYP6CB1* could not metabolise pyrethroids [46] although it could metabolise other insecticides. *CYP9J26* (AAEL014609) gene was among the top up-regulated detoxification gene which has also been reported in Cuba, Thailand and Grand Cayman [30] and has also been functionally validated to confer pyrethroid resistance [46].

Overall, several P450 genes belonging to the CYP9 family were over-expressed in the control- versus susceptible across Malaysia including two transcripts of *CYP9J27*, *CYP9J26*, *CYP9J28*, *CYP9M6* while only few cytochrome P450s from the CYP6 family (*CYP6P12*, *CYP6BB2*) were over-expressed and usually at lower fold change. This is further supported by previous studies worldwide showing that contrary to *Anopheles* mosquitoes, genes from the CYP9 family play a more important role than those from the CYP6 family in insecticide resistance in *Ae*. *aegypti* [7, 26, 30, 48, 49]. This was different than the *Aedes albopictus* transcription analyses performed with samples collected from same locations at the same time as more P450 genes belonging to the CYP6 family were over-expressed in the C-S comparison of *Ae*. *albopictus* samples in Malaysia including *CYP6N3*, *CYP6P12*, *CYP6Z6*, CYP6AG6, while only few cytochrome P450s from the CYP9 family were over-expressed [42].

Interestingly, the top most commonly over-expressed gene was the anionic-trypsin which is found in the midgut of mosquitoes and shown to hydrolyse proteins after blood meals. This serine proteinase is found to be over-expressed in deltamethrin resistant strain of *Culex pipiens pallens* from China [50].

Few glutathione S-transferases were detected compared to cytochrome P450s despite the very high DDT resistance notably in KL. The PBO synergist assay previously indicated a recovery of susceptibility from 0 to 35% in KL for DDT [10]. The low expression of GSTs notably that of the known DDT metaboliser *GSTe2* (FC = 4.5) [42, 45] suggests that knockdown resistance could be responsible for most of the remaining 65% loss of DDT susceptibility. Similar assessment of pyrethroids shows a recovery of susceptibility after PBO assay from 1% to 26% for permethrin and from 0% to 71% for deltamethrin. This suggests that metabolic resistance through P450 up-regulation is more important for deltamethrin than permethrin resistance and *kdr* playing a more important role for permethrin than deltamethrin. This will be in line with the higher correlation previously observed between permethrin and F1534C genotypes than with deltamethrin [10]. However, the molecular docking predicted CYP9J27 to bind and metabolise pyrethroids and DDT, especially KL model, compared to NO model to which DDT binds unproductively indicating lack of affinity and activity towards this organochlorine insecticide by NO strain.

Because PBO assays with bendiocarb also revealed a nearly full recovery of the susceptibility to this insecticide [10] it is likely that some of the cytochrome P450 genes detected in this study are responsible for this resistance although future functional characterization will identify the specific genes. Of course, with the exception of NO model, docking analyses with CYP9J27-616 models predicted productive binding and good affinity towards bendiocarb suggesting the ability to metabolise this carbamate insecticide. The possible role of P450s in carbamate resistance will explain the low expression of carboxylesterase genes observed in this study and suggests an absence of any *Ace-1* mutations as previously reported [49].

The higher polymorphism level of *CYP9J27-616* gene across Malaysian samples (apart from JB) (Table 2) suggests of little directional selection pressure favouring a specific SNP or amino acid change is acting on this gene in Malaysia despite it consistent over-expression. This suggests that *CYP9J27* potential role in the resistance if confirmed would be through a mechanism involving genetic variation in the regulatory regions such as promoter beside potential variation in the coding sequence. This is similar to cases of some P450 genes in other mosquito species for which despite a high over-expression in resistant individuals, no significant association has been observed between polymorphism level and resistance phenotype, such as *CYP6M7* in the malaria vector *An*. *funestus* [24]. Further analysis of these regulatory regions could help detect the specific genomic changes driving resistance through *CYP9J27*. Nevertheless, the lowest binding affinity from docking exhibited by the *CYP9J27* allele from the susceptible NO strain compared to those from Malaysian could suggest that allelic variation could also be playing a role in the resistance observed as recently demonstrated in the African malaria vector *An*. *funestus* [25]. Interestingly, negative values of neutrality tests Tajima’s, and Fu and Li’s observed for *CYP9J27* suggest either recent demographic expansion or background selection [35] on this gene across *Ae*. *aegypti* Malaysian population.

## Conclusion

This study revealed that the cytochrome P450 genes are associated in insecticide resistance in *Ae*. *aegypti* populations across Malaysia. However, further functional characterization work using either transgenic expression in *Drosophila* flies or recombinant enzymes expression in *Escherichia coli* coupled with metabolic assay has to be done to confirm the exact contribution of these candidate genes in the resistance profile. An alternative to pyrethroid is the organophosphate, malathion since all of the populations are susceptible to this insecticide [10].This and other proper resistance management strategies should be adopted to reverse the spread and evolution of this resistance problem in Malaysia before it compromises control programmes.

## Supporting Information

S1 Fig Schematic representation of microarray design. Green arrows refer to Cy-3 dye and red arrows refer to Cy-5 dye.
(PPTX)

S2 Fig Gene Ontology enrichment analysis of common genes in Penang, Kuala Lumpur and Kota Bharu. (PPTX)

S3 Fig Gene Ontology enrichment analysis of common genes in Kuala Lumpur. (PPTX)

S4 Fig Gene Ontology enrichment analysis of common genes in Penang. (PPTX)

S5 Fig Gene Ontology enrichment analysis of common genes in Khota Bharu. (PPTX)

S6 Fig Binding conformation of deltamethrin in (A) KL, (B) JB, (C) PG and (D) NO CYP9J27 models. Distances between the probable sites of attack on deltamethrin and heme iron are annotated.
(PPTX)

S7 Fig Binding conformation of DDT in (A) KL, (B) JB, (C) PG and (D) NO CYP9J27 models. Distances between the probable sites of attack on DDT and the heme iron are annotated.
(PPTX)

S8 Fig Binding conformation of bendiocarb in (A) KL, (B) JB, (C) PG and (D) NO CYP9J27 models. Distances between the probable sites of attack on bendiocarb and the heme iron are annotated.
(PPTX)

S9 Fig Binding conformation of (A) permethrin, (B) deltamethrin, (C) DDT and (D) bendiocarb in KB CYP9J27 model. Distances between the probable sites of attack on various insecticides and the heme iron are annotated.
(PPTX)

S1 Table Primers used for qRT-PCR for microarray candidate genes validation. (DOCX)

S2 Table Primer design for sequencing of *Aedes aegypti* CYP9M4 and CYP9J27. (DOCX)

S3 Table Top 50 commonly up-regulated probes in all three locations in comparison with susceptible New Orleans strain. FC = fold change. (p = 0.01).
(DOCX)

S4 Table Top 20 commonly down-regulated probes in all three locations in comparison with susceptible New Orleans strain. FC = fold change. (p = 0.01).
(DOCX)

S5 Table Probes from detoxification genes & genes linked with resistance down-regulated in all three locations in comparison with susceptible New Orleans strain. FC = fold change. (p = 0.01).
(DOCX)

S6 Table Binding parameters of the productive poses of permethrin, deltamethrin, DDT and bendiocarb in the active sites of various *Ae*. *aegypti* CYP9J27 models. (DOCX)
