# Supplementary figures and images for: Pyrethroid Resistance in Malaysian Populations of Dengue Vector Aedes aegypti Is Mediated by CYP9 Family of Cytochrome P450 Genes
Source: PLoS Negl Trop Dis. 2017 Jan 23;11(1):e0005302. doi: 10.1371/journal.pntd.0005302 (PMC5289618; doi:10.1371/journal.pntd.0005302)

## Slide 1
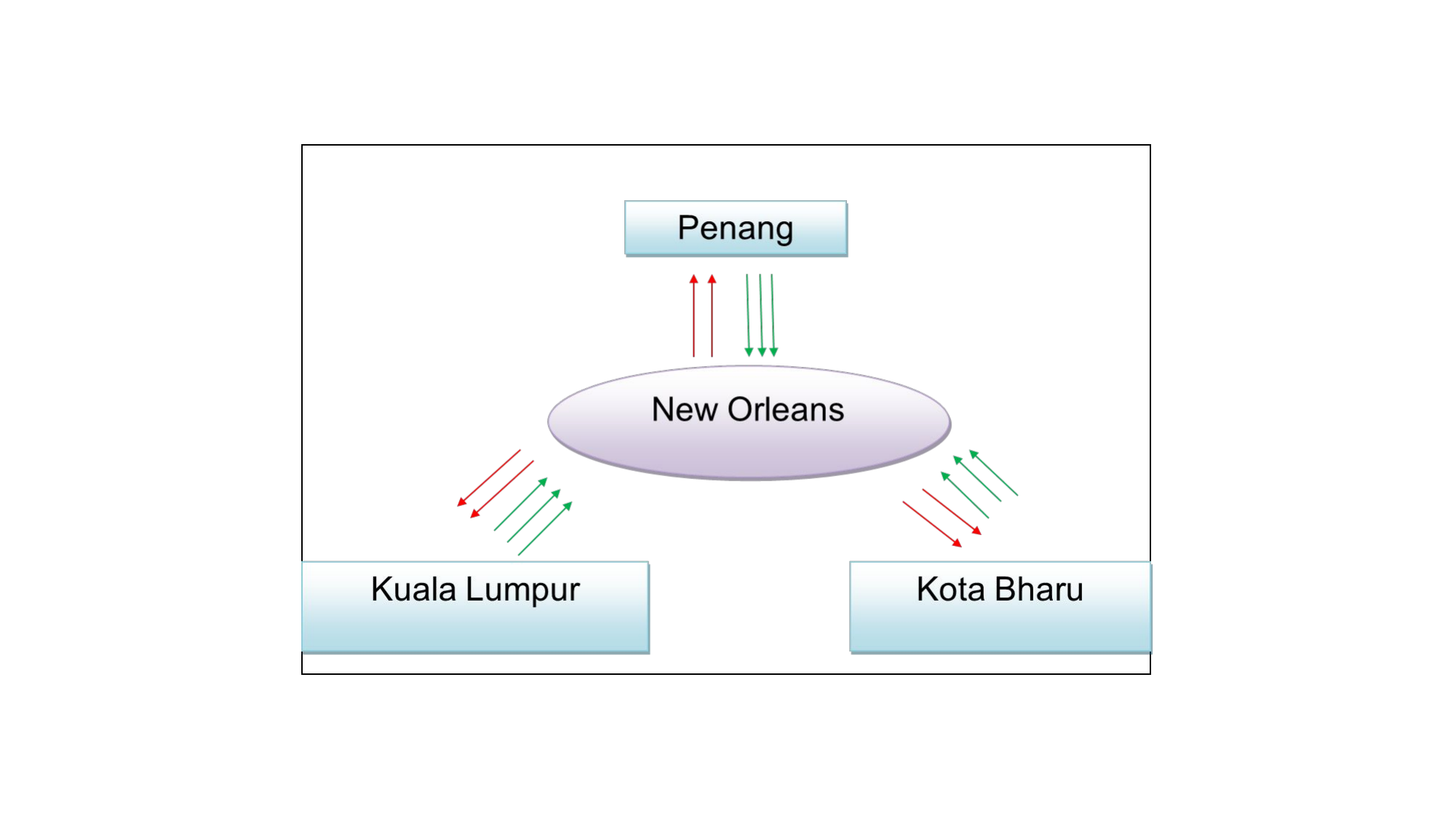

Supplement: S1 Fig — Green arrows refer to Cy-3 dye and red arrows refer to Cy-5 dye. (PPTX) [file pntd.0005302.s001.pptx]

## Slide 1
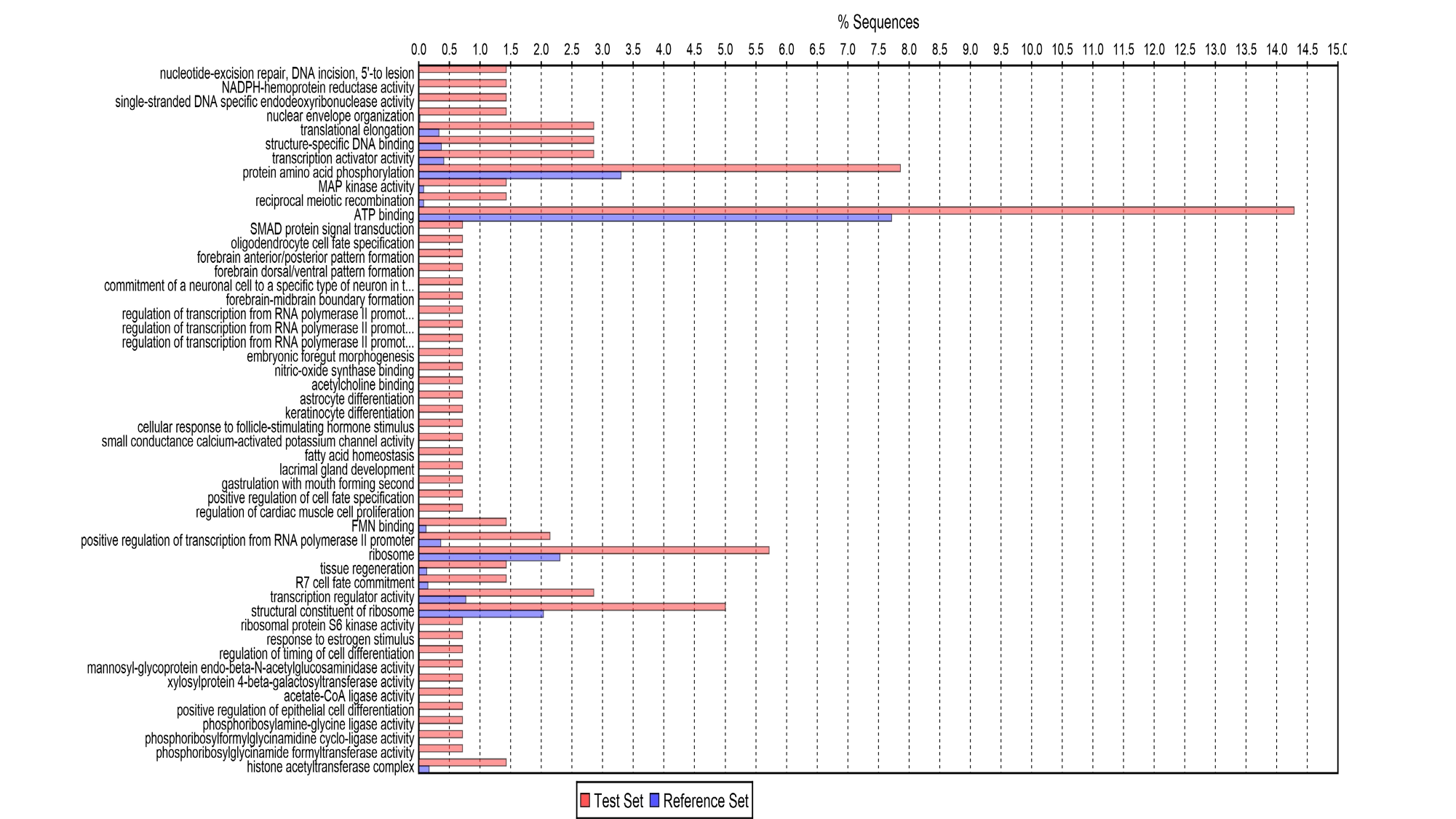

Supplement: S2 Fig — (PPTX) [file pntd.0005302.s002.pptx]

## Slide 1
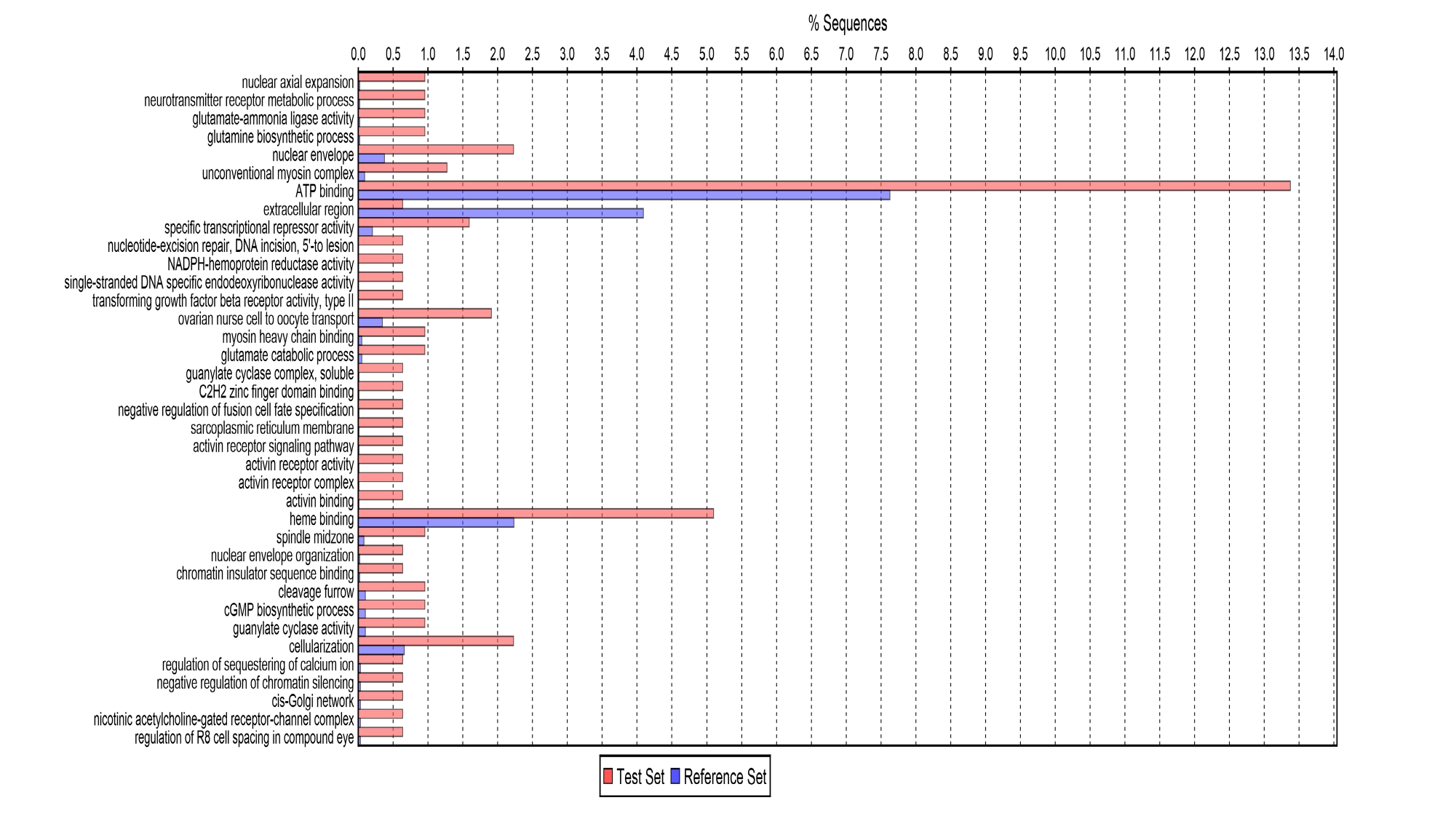

Supplement: S3 Fig — (PPTX) [file pntd.0005302.s003.pptx]

## Slide 1
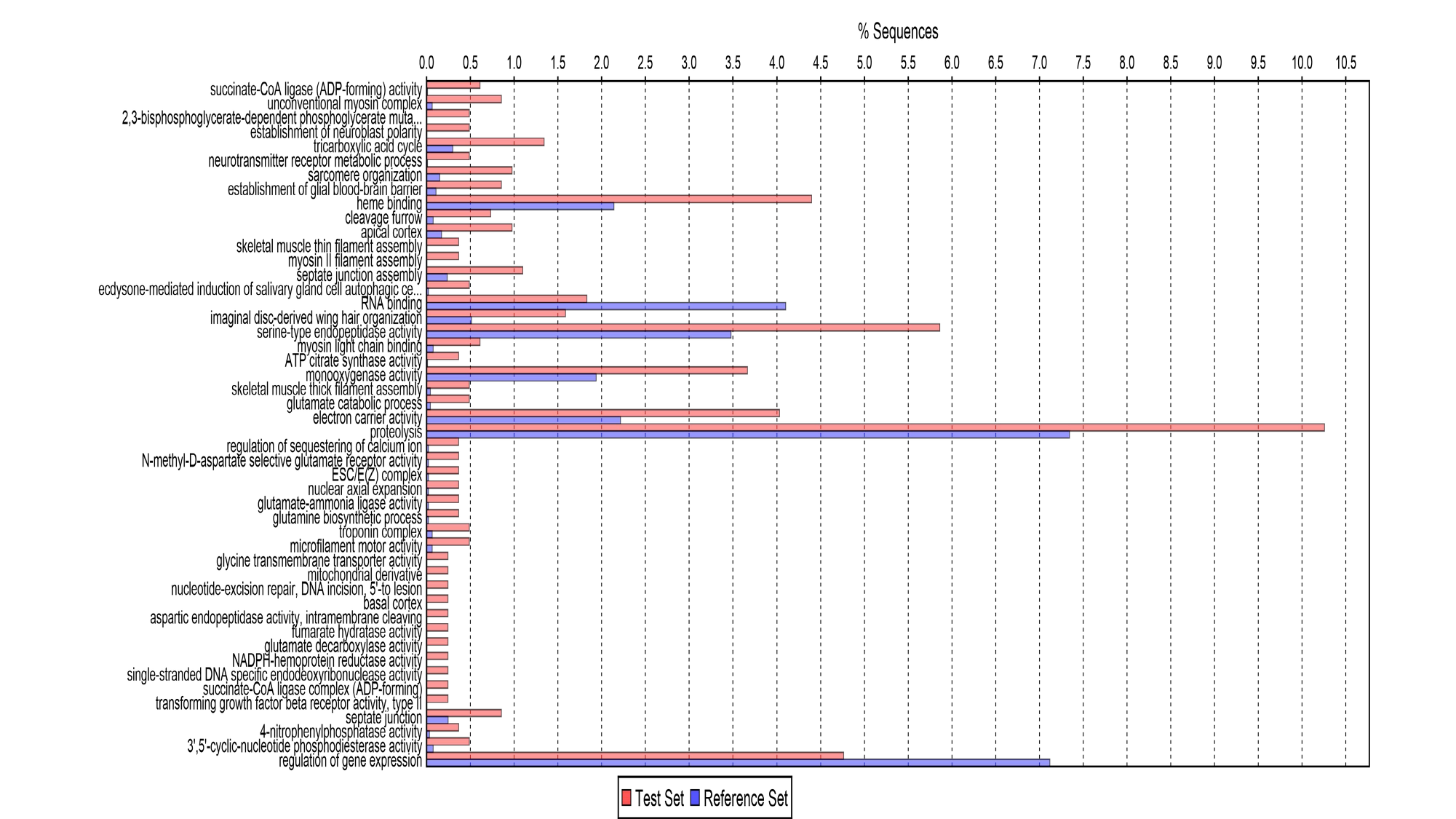

Supplement: S4 Fig — (PPTX) [file pntd.0005302.s004.pptx]

## Slide 1
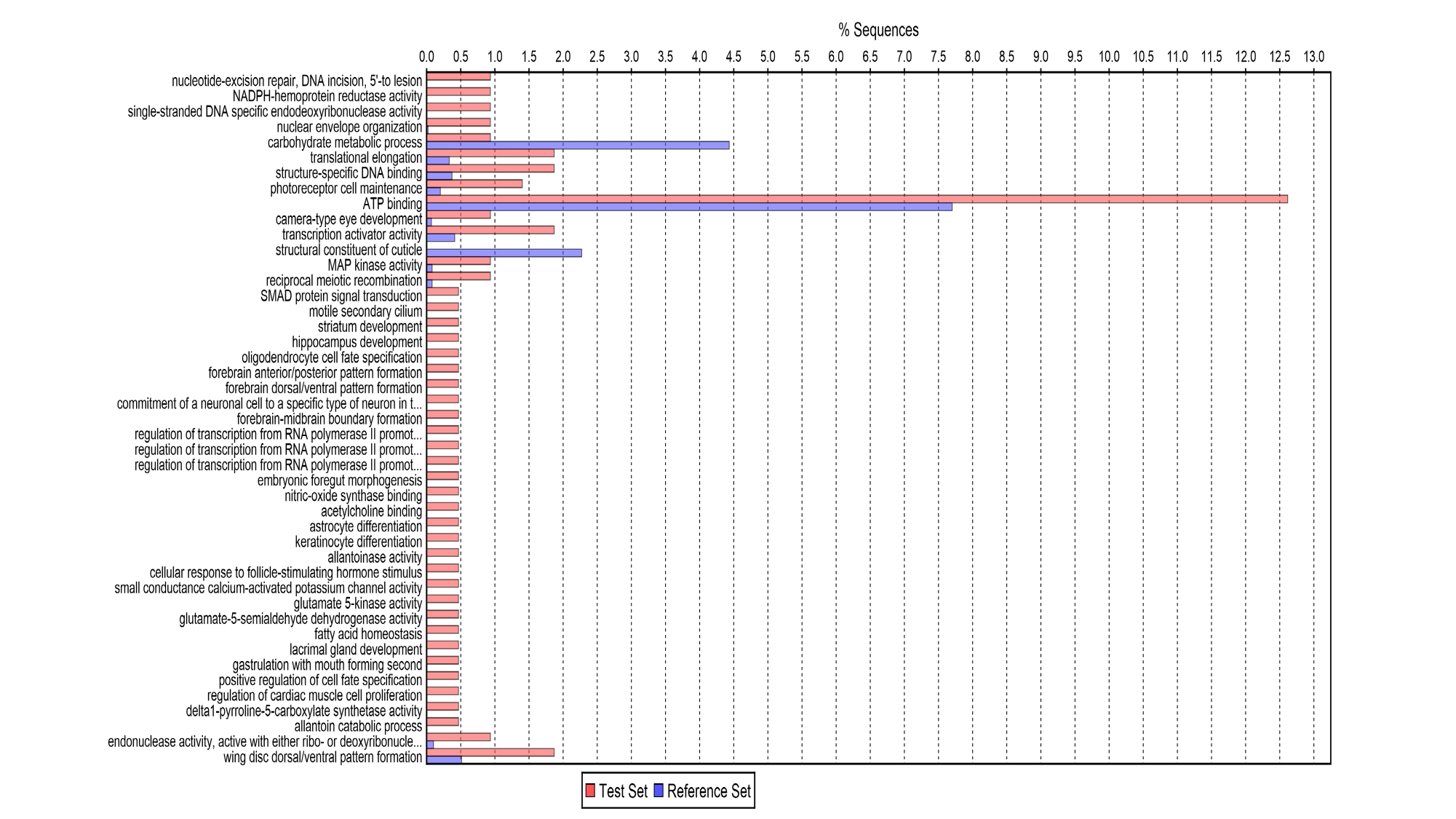

GO Terms

Supplement: S5 Fig — (PPTX) [file pntd.0005302.s005.pptx]

## Slide 1
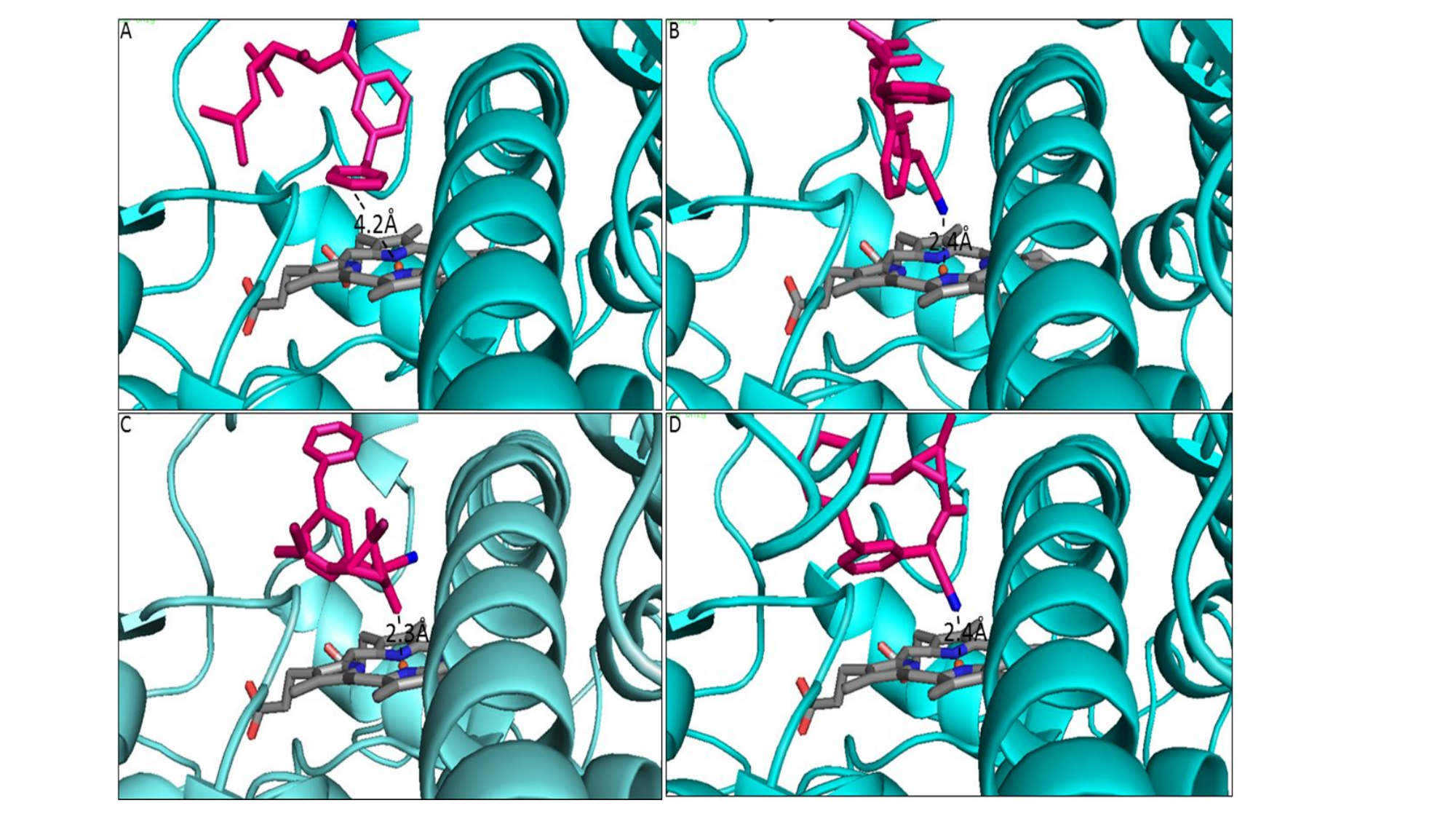

Supplement: S6 Fig — Binding conformation of deltamethrin in (A) KL, (B) JB, (C) PG and (D) NO CYP9J27 models. Distances between the probable sites of attack on deltamethrin and heme iron are annotated. (PPTX) [file pntd.0005302.s006.pptx]

## Slide 1
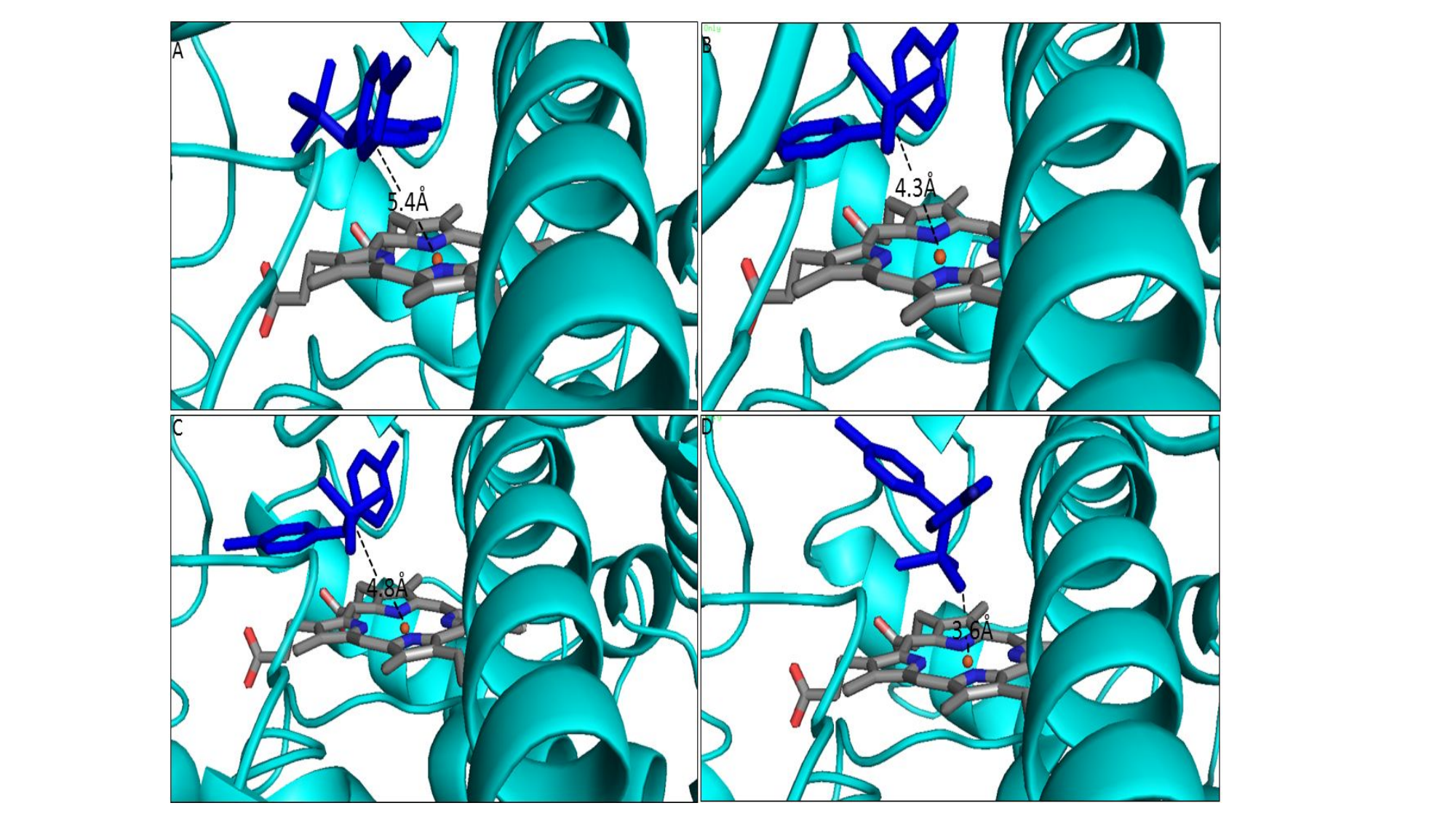

Supplement: S7 Fig — Binding conformation of DDT in (A) KL, (B) JB, (C) PG and (D) NO CYP9J27 models. Distances between the probable sites of attack on DDT and the heme iron are annotated. (PPTX) [file pntd.0005302.s007.pptx]

## Slide 1
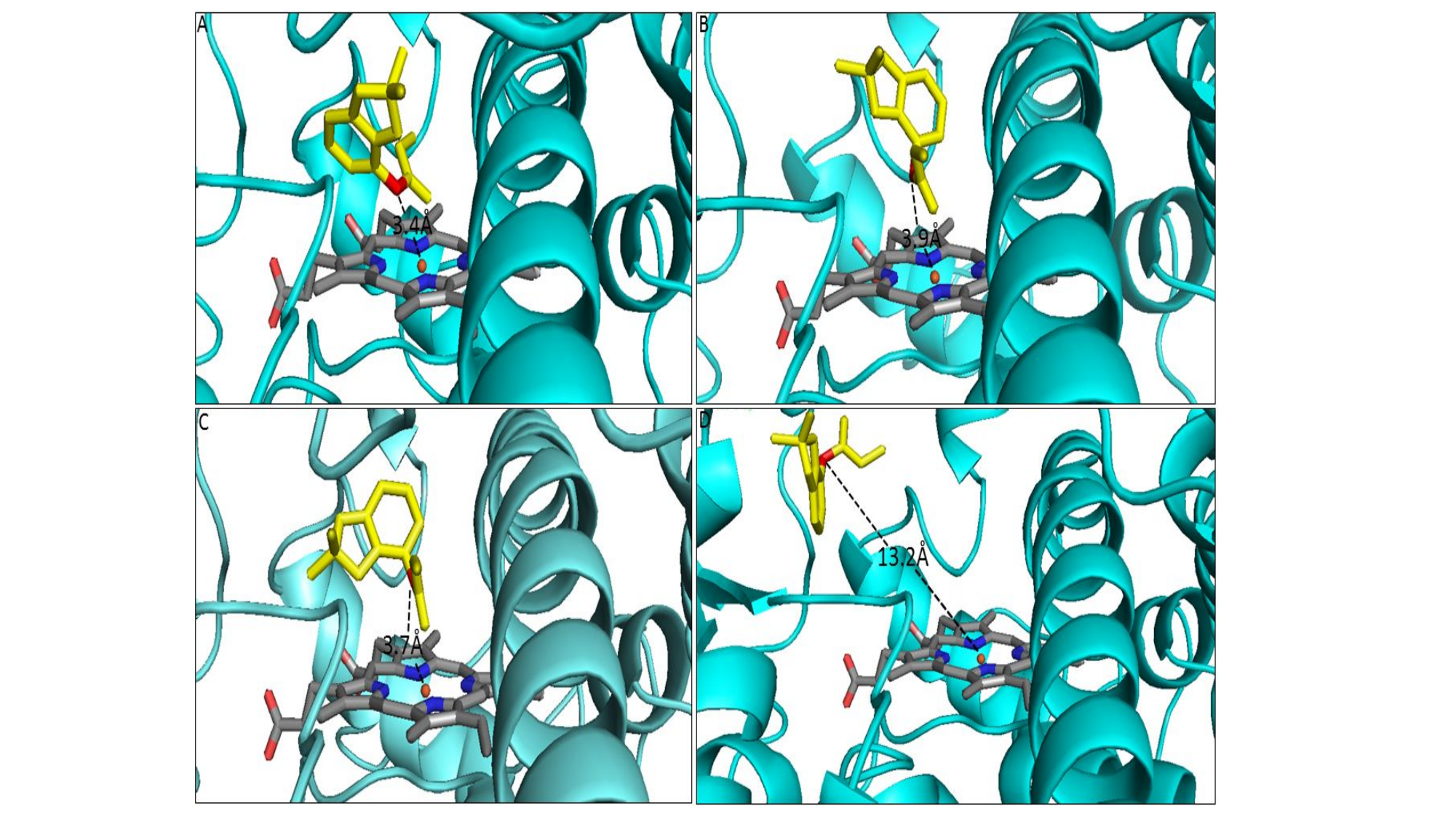

Supplement: S8 Fig — Binding conformation of bendiocarb in (A) KL, (B) JB, (C) PG and (D) NO CYP9J27 models. Distances between the probable sites of attack on bendiocarb and the heme iron are annotated. (PPTX) [file pntd.0005302.s008.pptx]

## Slide 1
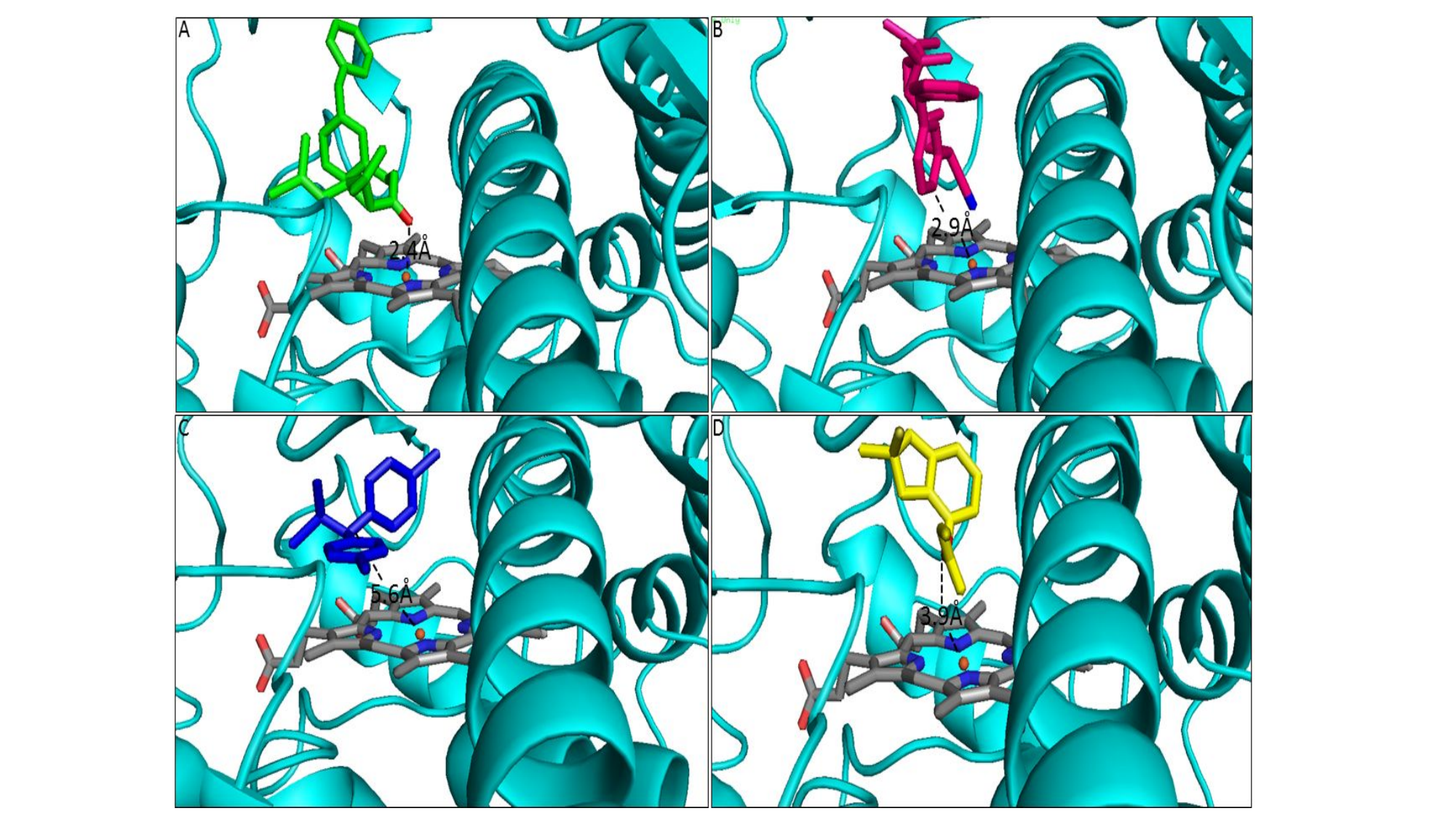

Supplement: S9 Fig — Binding conformation of (A) permethrin, (B) deltamethrin, (C) DDT and (D) bendiocarb in KB CYP9J27 model. Distances between the probable sites of attack on various insecticides and the heme iron are annotated. (PPTX) [file pntd.0005302.s009.pptx]
