# Supplementary material for: Pyrethroid Resistance in Malaysian Populations of Dengue Vector Aedes aegypti Is Mediated by CYP9 Family of Cytochrome P450 Genes
Source: PLoS Negl Trop Dis. 2017 Jan 23;11(1):e0005302. doi: 10.1371/journal.pntd.0005302 (PMC5289618; doi:10.1371/journal.pntd.0005302)
Supplement: S1 Table — (DOCX) [file pntd.0005302.s010.docx]

| **Primer Name** | **Sequence** | **Product size**  **(base pairs)** | **SYBR Standards (RSq)** | **Efficiency (%)** |
| --- | --- | --- | --- | --- |
| qCYP6CB1 F | TGAAATCGAGCTGGATCCTT | 130 | 0.995 | 90.0 |
| qCYP6CB1 R | CTCCTAATGCTTCCATTACTCAA |  |  |  |
| qAAEL006727 F | CTACCAGTGCGATCAAGCAG | 127 | 0.998 | 91.7 |
| qAAEL006727 R | AATCCTTTGCGTTTCACCTG |  |  |  |
| qAAEL013623 F | TGGCCAACCTTCCTCTGTAA | 123 | 1.000 | 92.4 |
| qAAEL013623 R | CCTGCTAATTGTTGTGCTTCA |  |  |  |
| qCYP9J26-607 F | CACGCTGCTGAAGTTTACGA | 150 | 0.976 | 99.3 |
| qCYP9J26-607 R | AAAATTTGGACAAAACCCTATTCA |  |  |  |
| qCYP9M4 F | GGTTGATCACGAAGGACGTT | 114 | 0.882 | 94.6 |
| qCYP9M4 R | CCTGCACGAACAAATGAATG |  |  |  |
| qCYP9J27 F | CACCGTTCAGGAGTCAGACA | 128 | 0.995 | 90.0 |
| qCYP9J27 R | TGAACATGGCACAGGTTGAT |  |  |  |
| qCYP9J26-609 F | TGCACCACGATCCACAGTAT | 109 | 0.997 | 90.7 |
| qCYP9J26-609 R | TACCAAACGGCAGATACGC |  |  |  |
| qTub-Aae F | CCGCACTCGAGAAGGATTAC | 131 | 0.996 | 92.2 |
| qTub-Aae R | GTGGTTCGGTTTGACTTCGT |  |  |  |
| qRPS7-Aae F | AAGGTCGACACCTTCACGTC | 131 | 0.986 | 90.1 |
| qRPS7-Aae R | CGCGCGCTCACTTATTAGAT |  |  |  |
