# Supplementary material for: Pyrethroid Resistance in Malaysian Populations of Dengue Vector Aedes aegypti Is Mediated by CYP9 Family of Cytochrome P450 Genes
Source: PLoS Negl Trop Dis. 2017 Jan 23;11(1):e0005302. doi: 10.1371/journal.pntd.0005302 (PMC5289618; doi:10.1371/journal.pntd.0005302)
Supplement: S2 Table — (DOCX) [file pntd.0005302.s011.docx]

| **Primer name** | **Sequence (5’-3’)** | **Targeted Gene** | **Product size**  **(base pairs)** |
| --- | --- | --- | --- |
| 9M4_UTR5_F | GATTCGCTACTCGCCACAAGTA | CYP9M4 | 22 |
| 9M4_intern_R | AACTCAACGACAGCCCTCTGACA | CYP9M4 | 23 |
| 9M4_UTR3_R | CGATTGTCATCATGAATCAGTTCAGT | CYP9M4 | 26 |
| 9J27_UTR5_F | AACTGCAAGGGACATGGAGG | CYP9J27 | 20 |
| 9J27_intern_R | GCTTCCGGTGAAGGCCG | CYP9J27 | 17 |
| 9J27_UTR3_R | TCCTATTCATCTCAGCTTCAACTCC | CYP9J27 | 25 |
