# Supplementary material for: Pyrethroid Resistance in Malaysian Populations of Dengue Vector Aedes aegypti Is Mediated by CYP9 Family of Cytochrome P450 Genes
Source: PLoS Negl Trop Dis. 2017 Jan 23;11(1):e0005302. doi: 10.1371/journal.pntd.0005302 (PMC5289618; doi:10.1371/journal.pntd.0005302)
Supplement: S3 Table — FC = fold change. (p = 0.01). (DOCX) [file pntd.0005302.s012.docx]

| **Probe Name** | **Systematic Name** | **Blast2GO Annotation** | **Kota Bharu vs NO** | | **Kuala Lumpur vs NO** | | **Penang vs NO** | |
| --- | --- | --- | --- | --- | --- | --- | --- | --- |
|  |  |  | **Absolute FC** | **Corrected p-value** | **Absolute FC** | **Corrected p-value** | **Absolute FC** | **Corrected p-value** |
| CUST_920_PI424980000 | AAEL013623-RA | anionic trypsin-2 | 43.86 | 0.044294 | 759.15 | 0.00108 | 581.43 | 2.67E-04 |
| CUST_3568_PI424980000 | AAEL011733-RA | transcription elongation regulator 1 | 56.06 | 0.036507 | 448.21 | 0.001285 | 255.18 | 3.55E-04 |
| CUST_6004_PI424980000 | AAEL009891-RA | isoform a | 136.08 | 0.001126 | 299.55 | 0.003494 | 167.27 | 0.001721 |
| CUST_8758_PI424980000 | AAEL000183-RA | hypothetical protein | 148.20 | 1.49E-04 | 269.77 | 0.001346 | 149.85 | 4.07E-04 |
| CUST_11373_PI424980000 | AAEL000888-RA | single stranded dna binding protein | 153.09 | 2.29E-04 | 232.52 | 0.001143 | 100.23 | 9.22E-04 |
| CUST_11374_PI424980000 | AAEL000888-RB | single stranded dna binding protein | 129.32 | 2.21E-04 | 210.12 | 1.65E-04 | 136.52 | 7.94E-04 |
| CUST_3849_PI424980000 | AAEL001806-RA | microsomal triglyceride transfer protein large subunit | 15.54 | 0.028302 | 177.68 | 0.003888 | 138.31 | 4.28E-04 |
| CUST_2177_PI424980000 | AAEL013693-RA | excision repair cross-complementing 1 ercc1 | 34.69 | 0.047021 | 163.73 | 0.001663 | 193.23 | 3.46E-04 |
| CUST_9198_PI424980000 | AAEL006727-RA | multisynthetase auxiliary | 238.54 | 3.45E-04 | 159.16 | 0.002556 | 185.03 | 1.73E-04 |
| CUST_9199_PI424980000 | AAEL006727-RB | multisynthetase auxiliary | 296.08 | 2.21E-04 | 148.03 | 0.002085 | 298.80 | 2.67E-04 |
| CUST_2020_PI424980000 | AAEL012726-RA | hypothetical protein | 148.57 | 2.21E-04 | 137.98 | 0.001687 | 98.50 | 3.26E-04 |
| CUST_2845_PI424980000 | AAEL009018-RA | cytochrome p450 (CYP6CB1) | 36.37 | 0.048806 | 124.69 | 0.001285 | 211.97 | 7.56E-04 |
| CUST_10987_PI424980000 | AAEL003672-RA | zinc finger protein | 40.78 | 0.003836 | 112.54 | 0.001635 | 126.80 | 2.69E-04 |
| CUST_5231_PI424980000 | AAEL011383-RA | mage protein | 28.97 | 0.04977 | 108.64 | 0.005995 | 106.99 | 3.90E-04 |
| CUST_2768_PI424980000 | AAEL010097-RA | exuperantia 2 | 17.00 | 0.04184 | 99.63 | 0.001143 | 109.67 | 1.40E-04 |
| CUST_2609_PI424980000 | AAEL015136-RA | niemann-pick type c- | 55.13 | 3.45E-04 | 97.61 | 0.001467 | 173.74 | 1.40E-04 |
| CUST_1955_PI424980000 | AAEL008525-RA | zinc finger imprinted 3 | 61.41 | 8.08E-04 | 91.20 | 0.001346 | 20.60 | 0.005476 |
| CUST_6355_PI424980000 | AAEL003679-RA | zinc finger protein | 28.29 | 7.33E-04 | 77.60 | 0.002124 | 42.45 | 8.56E-04 |
| CUST_11261_PI424980000 | AAEL004545-RA | cas1 domain-containing protein 1-like | 16.11 | 0.02661 | 66.05 | 0.001053 | 70.17 | 5.16E-04 |
| CUST_7094_PI424980000 | AAEL012673-RA | ubiquitin conjugating enzyme 7 interacting protein | 24.00 | 0.004167 | 65.55 | 0.003194 | 45.66 | 0.001165 |
| CUST_3521_PI424980000 | AAEL010555-RA | sterol regulatory element-binding protein 1 | 55.42 | 2.94E-04 | 65.14 | 0.003143 | 78.23 | 8.14E-04 |
| CUST_2969_PI424980000 | AAEL000321-RA | acetyl-coa synthetase | 25.12 | 9.29E-04 | 54.66 | 0.002708 | 29.79 | 0.004955 |
| CUST_5474_PI424980000 | AAEL007632-RA | myosin light chain kinase | 8.37 | 0.008264 | 54.57 | 0.002445 | 35.71 | 0.001564 |
| CUST_11672_PI424980000 | AAEL010222-RB | gata transcription factor | 29.35 | 3.07E-04 | 47.12 | 0.002157 | 18.17 | 7.42E-04 |
| CUST_13241_PI424980000 | AAEL012487-RA | hypothetical protein | 9.45 | 0.037488 | 46.50 | 0.002874 | 22.19 | 0.006043 |
| CUST_12609_PI424980000 | AAEL005786-RA | isoform a | 16.33 | 2.94E-04 | 45.41 | 0.005995 | 20.72 | 7.51E-04 |
| CUST_2242_PI424980000 | AAEL003455-RA | isoform a | 16.12 | 0.047723 | 42.08 | 0.00754 | 64.79 | 9.22E-04 |
| CUST_13231_PI424980000 | AAEL002446-RA | isoform b | 16.05 | 0.034975 | 40.15 | 0.003882 | 29.95 | 3.69E-04 |
| CUST_13389_PI424980000 | AAEL012357-RB | hemolymph protein | 11.48 | 0.048806 | 38.26 | 0.003208 | 69.58 | 0.002092 |
| CUST_7459_PI424980000 | AAEL013110-RA | isoform a | 17.08 | 0.002495 | 37.16 | 0.003682 | 14.45 | 0.002239 |
| CUST_12920_PI424980000 | AAEL003700-RA | zinc finger protein | 17.07 | 9.29E-04 | 36.87 | 0.002139 | 43.03 | 6.70E-04 |
| CUST_6811_PI424980000 | AAEL014583-RA | 60s acidic ribosomal protein p2 | 23.36 | 0.002899 | 34.51 | 0.00108 | 38.49 | 1.44E-04 |
| CUST_1318_PI424980000 | AAEL005745-RA | gpcr class a orphan receptor 18 (agap005002-pb) | 19.49 | 5.38E-04 | 33.65 | 0.003315 | 14.15 | 0.003402 |
| CUST_6812_PI424980000 | AAEL014583-RB | 60s acidic ribosomal protein p2 | 22.10 | 0.008264 | 33.58 | 0.00108 | 40.38 | 1.34E-04 |
| CUST_11048_PI424980000 | AAEL010247-RA | cg5913 cg5913-pa | 14.88 | 0.002906 | 33.27 | 0.00108 | 37.73 | 7.03E-04 |
| CUST_11671_PI424980000 | AAEL010222-RA | gata transcription factor | 26.73 | 3.45E-04 | 32.81 | 0.001309 | 13.35 | 6.29E-04 |
| CUST_658_PI424980000 | AAEL012357-RA | hemolymph protein | 14.37 | 0.047514 | 32.40 | 0.005494 | 49.00 | 7.69E-04 |
| CUST_4722_PI424980000 | AAEL004530-RA | zinc finger protein | 12.69 | 0.030913 | 31.97 | 0.003494 | 41.14 | 3.26E-04 |
| CUST_7952_PI424980000 | AAEL009828-RA | vacuolar fusion protein ccz1 homolog | 6.58 | 0.017094 | 31.97 | 0.003045 | 9.94 | 0.001053 |
| CUST_6813_PI424980000 | AAEL014583-RC | 60s acidic ribosomal protein  p2 | 21.47 | 0.002906 | 30.30 | 0.001309 | 38.47 | 1.04E-04 |
| CUST_2975_PI424980000 | AAEL009076-RA | nadh dehydrogenase subunit 4 | 13.26 | 0.011725 | 28.77 | 0.004492 | 35.40 | 3.07E-04 |
| CUST_5654_PI424980000 | AAEL012983-RA | king isoform b | 13.54 | 0.022381 | 28.57 | 0.003875 | 28.33 | 8.37E-04 |
| CUST_7348_PI424980000 | AAEL000229-RA | sialokinin i preproprotein | 75.55 | 8.33E-04 | 28.20 | 0.022194 | 114.84 | 3.10E-04 |
| CUST_6523_PI424980000 | AAEL007147-RA | hypothetical protein | 7.44 | 0.026524 | 27.16 | 0.00593 | 18.52 | 6.50E-04 |
| CUST_11049_PI424980000 | AAEL010247-RB | family with sequence similarity member b-like | 11.32 | 0.002906 | 25.63 | 0.002139 | 21.68 | 0.001045 |
| CUST_3982_PI424980000 | AAEL004187-RA | gpcr class a orphan receptor 18 (agap005002-pa) | 15.42 | 7.72E-04 | 24.97 | 0.001687 | 8.45 | 0.011122 |
| CUST_11072_PI424980000 | AAEL007984-RA | hypothetical protein | 8.06 | 0.02135 | 24.45 | 0.006746 | 14.29 | 0.002093 |
| CUST_9292_PI424980000 | AAEL003349-RA | nadph-cytochrome p450 reductase | 6.63 | 0.044205 | 24.23 | 0.004212 | 8.85 | 1.22E-04 |
| CUST_6584_PI424980000 | AAEL013215-RA | sulfonylurea receptor abc transporter | 10.65 | 0.002906 | 24.01 | 0.00503 | 4.57 | 0.009755 |
| CUST_13373_PI424980000 | AAEL003052-RA | isoform a | 5.81 | 0.0105 | 22.96 | 0.004212 | 5.88 | 0.002497 |
