# Supplementary material for: Pyrethroid Resistance in Malaysian Populations of Dengue Vector Aedes aegypti Is Mediated by CYP9 Family of Cytochrome P450 Genes
Source: PLoS Negl Trop Dis. 2017 Jan 23;11(1):e0005302. doi: 10.1371/journal.pntd.0005302 (PMC5289618; doi:10.1371/journal.pntd.0005302)
Supplement: S4 Table — FC = fold change. (p = 0.01). (DOCX) [file pntd.0005302.s013.docx]

| **Probe Name** | **Systematic Name** | **Blast2GO Annotation** | **Kota Bharu vs NO** | | **Kuala Lumpur vs NO** | | **Penang vs NO** | |
| --- | --- | --- | --- | --- | --- | --- | --- | --- |
|  |  |  | **Absolute FC** | **Corrected p-value** | **Absolute FC** | **Corrected p-value** | **Absolute FC** | **Corrected p-value** |
| CUST_3200_PI424980000 | AAEL013569-RA | domain-containing protein cg6693 | 31.21 | 0.02558 | 33.07 | 0.046552 | 53.72 | 2.35E-04 |
| CUST_12358_PI424980000 | AAEL001887-RB | glutamine synthetase 2 | 4.02 | 0.002906 | 18.63 | 0.019508 | 12.71 | 0.001995 |
| CUST_12357_PI424980000 | AAEL001887-RA | glutamine synthetase 2 | 3.79 | 4.27E-04 | 13.70 | 0.025112 | 16.18 | 0.001403 |
| CUST_7707_PI424980000 | AAEL000535-RA | galactose-specific c-type | 7.07 | 0.019551 | 12.85 | 0.003315 | 12.90 | 2.13E-04 |
| CUST_3015_PI424980000 | AAEL004397-RA | ankyrin repeat | 2.70 | 1.68E-04 | 11.64 | 0.046495 | 31.81 | 0.001583 |
| CUST_11275_PI424980000 | AAEL001828-RA | hypothetical protein | 2.52 | 0.009108 | 9.30 | 0.037338 | 16.73 | 6.28E-04 |
| CUST_4719_PI424980000 | AAEL010221-RA | gata transcription factor gatad | 3.38 | 0.029791 | 7.02 | 0.012952 | 16.43 | 0.001281 |
| CUST_1302_PI424980000 | AAEL007347-RA | serine protease | 2.35 | 0.002536 | 5.84 | 0.017178 | 5.67 | 0.005945 |
| CUST_3177_PI424980000 | AAEL001274-RA | hypothetical protein | 5.42 | 0.004642 | 5.79 | 0.003556 | 8.13 | 3.69E-04 |
| CUST_5528_PI424980000 | AAEL006280-RA | juvenile hormone acid methyltransferase | 2.05 | 0.00194 | 5.56 | 0.026461 | 5.28 | 0.00248 |
| CUST_5946_PI424980000 | AAEL008224-RA | hypothetical protein | 2.17 | 0.044052 | 5.53 | 0.011167 | 26.36 | 0.001065 |
| CUST_22_PI424980000 | AAEL012769-RA | cytochrome p450 (CYP325M2) | 2.95 | 0.006449 | 5.36 | 0.011548 | 6.39 | 9.43E-04 |
| CUST_11250_PI424980000 | AAEL004342-RA | odorant-binding protein | 3.68 | 0.019184 | 5.11 | 0.038264 | 13.82 | 4.95E-04 |
| CUST_2153_PI424980000 | AAEL015650-RA | isoform a | 3.37 | 0.009584 | 4.97 | 0.009499 | 7.92 | 2.69E-04 |
| CUST_8546_PI424980000 | AAEL012850-RA | isoform a | 3.50 | 0.012497 | 4.86 | 0.019799 | 8.60 | 2.67E-04 |
| CUST_1674_PI424980000 | AAEL015468-RA | hypothetical protein | 2.61 | 0.021134 | 4.79 | 0.008665 | 3.66 | 0.00596 |
| CUST_10633_PI424980000 | AAEL002447-RA | hypothetical protein | 2.12 | 0.013594 | 4.67 | 0.011003 | 3.11 | 7.97E-04 |
| CUST_7240_PI424980000 | AAEL014163-RA | serine protease | 2.51 | 0.017204 | 4.64 | 0.0141 | 4.88 | 0.003521 |
| CUST_6420_PI424980000 | AAEL006466-RA | chondroitin synthase | 2.40 | 0.013921 | 4.51 | 0.003494 | 71.29 | 9.62E-04 |
| CUST_2695_PI424980000 | AAEL009825-RA | 60s ribosomal protein l13a | 2.12 | 0.002906 | 4.14 | 0.013701 | 18.05 | 3.02E-04 |
