# Supplementary material for: Pyrethroid Resistance in Malaysian Populations of Dengue Vector Aedes aegypti Is Mediated by CYP9 Family of Cytochrome P450 Genes
Source: PLoS Negl Trop Dis. 2017 Jan 23;11(1):e0005302. doi: 10.1371/journal.pntd.0005302 (PMC5289618; doi:10.1371/journal.pntd.0005302)
Supplement: S5 Table — FC = fold change. (p = 0.01). (DOCX) [file pntd.0005302.s014.docx]

| **Probe Name** | **Systematic Name** | **Blast2GO Annotation** | **Kota Bharu vs NO** | | **Kuala Lumpur vs NO** | | **Penang vs NO** | |
| --- | --- | --- | --- | --- | --- | --- | --- | --- |
|  |  |  | **Absolute FC** | **Corrected p-value** | **Absolute FC** | **Corrected p-value** | **Absolute FC** | **Corrected p-value** |
| **Common to all three locations** | | | | | | | | |
| CUST_22_PI424980000 | AAEL012769-RA | cytochrome p450 (CYP325M2) | 2.95 | 0.006449 | 5.36 | 0.011548 | 6.39 | 9.43E-04 |
| CUST_12428_PI424980000 | AAEL015476-RA | cytochrome p450 (as CYP325F2 in *An. gambiae*) | 2.35 | 7.53E-04 |  |  | 4.41 | 0.005393 |
| CUST_18_PI424980000 | AAEL012766-RA | cytochrome p450 (CYP325G2) | 2.17 | 0.017221 |  |  | 13.90 | 3.65E-04 |
| **Common to KB and PG but not KL** | | | | | | | | |
| CUST_4119_PI424980000 | AAEL015475-RA | cytochrome p450 (as CYP325C2  in *An. gambiae*) | |  | 4.39 | 0.003937 | 15.83 | 0.001003 |
| CUST_55_PI424980000 | AAEL014019-RB | cytochrome p450 (CYP4J16) | |  | 4.09 | 0.007248 | 6.33 | 0.004038 |
| CUST_128_PI424980000 | AAEL012761-RA | cytochrome p450 (CYP325T2) | |  | 3.37 | 0.00709 | 9.06 | 7.90E-04 |
| **KL only** | | | | | | | | |
| CUST_127_PI424980000 | AAEL000325-RA | cytochrome p450 (as CYP325C3 in *An. gambiae*) | | | 3.48 | 0.010751 |  |  |
| CUST_11713_PI424980000 | AAEL005375-RA | glucosyl glucuronosyl transferases | | | 2.07 | 0.024392 |  |  |
| CUST_2203_PI424980000 | AAEL009298-RA | n-acetylneuraminate lyase | | | 2.61 | 0.009488 |  |  |
| CUST_2240_PI424980000 | AAEL012764-RA | glycine n-methyltransferase | | | 2.60 | 0.008627 |  |  |
| CUST_54_PI424980000 | AAEL013554-RA | cytochrome p450 (CYP4J14) | |  | 2.58 | 0.006615 |  |  |
| CUST_133_PI424980000 | AAEL013556-RA | cytochrome p450 (CYP4J15) | |  | 2.26 | 0.008072 |  |  |
| **PG only** | | | | | | | | |
| CUST_32_PI424980000 | AAEL005696-RA | cytochrome p450 (CYP325X2) | |  |  |  | 26.97 | 1.72E-04 |
| CUST_104_PI424980000 | AAEL014615-RA | cytochrome p450 (CYP9J23) | |  |  |  | 12.18 | 0.003133 |
| CUST_175_PI424980000 | AAEL007954-RA | glutathione-s-transferase (GSTE1) | | |  |  | 8.43 | 0.002579 |
| CUST_25_PI424980000 | AAEL012770-RA | cytochrome p450 (CYP32N1) | |  |  |  | 6.42 | 4.28E-04 |
| CUST_136_PI424980000 | AAEL014680-RA | cytochrome p450 (CYP6CA1) | |  |  |  | 6.32 | 2.93E-04 |
| CUST_68_PI424980000 | AAEL017539-RA | cytochrome p450 (CYP6BY1) | |  |  |  | 5.85 | 5.01E-04 |
| CUST_83_PI424980000 | AAEL009133-RA | cytochrome p450 (CYP6N14) | |  |  |  | 4.77 | 0.004511 |
| CUST_138_PI424980000 | AAEL009122-RA | cytochrome p450 (as CYP6N2  in *An. gambiae*) | |  |  |  | 4.53 | 0.005026 |
| CUST_53_PI424980000 | AAEL013555-RA | cytochrome p450 (CYP4J13) | |  |  |  | 4.31 | 2.81E-04 |
| CUST_56_PI424980000 | AAEL014019-RA | cytochrome p450 (CYP4J16) | |  |  |  | 4.27 | 5.49E-04 |
| CUST_117_PI424980000 | AAEL002067-RA | cytochrome p450 (as CYP15B1  in *An. gambiae*) | |  |  |  | 3.59 | 8.86E-04 |
| CUST_342_PI424980000 | AAEL014019-RA | cytochrome p450 (CYP4J16) | |  |  |  | 3.58 | 0.00418 |
| CUST_139_PI424980000 | AAEL003748-RA | cytochrome p450 (CYP9AE1) | |  |  |  | 3.47 | 2.81E-04 |
| CUST_49_PI424980000 | AAEL003380-RA | cytochrome p450 (CYP4H28) | |  |  |  | 3.37 | 7.37E-04 |
| CUST_3780_PI424980000 | AAEL014830-RA | cytochrome p450 (as CYP4J10  in *An. gambiae*) | |  |  |  | 3.25 | 9.39E-04 |
| CUST_2203_PI424980000 | AAEL009298-RA | n-acetylneuraminate lyase | | |  |  | 2.87 | 0.001276 |
| CUST_89_PI424980000 | AAEL009120-RA | cytochrome p450 (CYP6S3) | |  |  |  | 2.82 | 8.19E-04 |
| CUST_118_PI424980000 | AAEL011463-RA | cytochrome p450 (as CYP302A1  in *An. gambiae*) | |  |  |  | 2.69 | 9.83E-04 |
| CUST_355_PI424980000 | AAEL008635-RA | abc transporter | |  |  |  | 2.67 | 4.52E-04 |
| CUST_39_PI424980000 | AAEL010154-RA | cytochrome p450 (CYP4AR2) | |  |  |  | 2.57 | 0.004548 |
| CUST_63_PI424980000 | AAEL015654-RA | cytochrome p450 (CYP6AG8) | |  |  |  | 2.57 | 0.001943 |
| CUST_54_PI424980000 | AAEL013554-RA | cytochrome p450 (CYP4J14) | |  |  |  | 2.56 | 3.26E-04 |
| CUST_125_PI424980000 | AAEL006058-RA | cytochrome p450 (CYP325Q2) | |  |  |  | 2.53 | 0.003763 |
| CUST_20_PI424980000 | AAEL005788-RA | cytochrome p450 (CYP325K3) | |  |  |  | 2.51 | 0.00114 |
| CUST_8_PI424980000 | AAEL015655-RA | cytochrome p450 (CYP302A1) | |  |  |  | 2.24 | 0.001686 |
| CUST_9_PI424980000 | AAEL014412-RA | cytochrome p450 (CYP304B2) | |  |  |  | 2.23 | 0.003468 |
| CUST_196_PI424980000 | AAEL002385-RA | Carboxylesterase (CCEAE3B) | |  |  |  | 2.23 | 5.35E-04 |
| CUST_2721_PI424980000 | AAEL008663-RA | aldo-keto reductase | |  |  |  | 2.20 | 0.009233 |
| CUST_82_PI424980000 | AAEL009137-RA | cytochrome p450 (CYP6N13) | |  |  |  | 2.18 | 0.001756 |
| CUST_85_PI424980000 | AAEL010158-RA | cytochrome p450 (CYP6N17) | |  |  |  | 2.16 | 0.008654 |
