# Supplementary material for: Pyrethroid Resistance in Malaysian Populations of Dengue Vector Aedes aegypti Is Mediated by CYP9 Family of Cytochrome P450 Genes
Source: PLoS Negl Trop Dis. 2017 Jan 23;11(1):e0005302. doi: 10.1371/journal.pntd.0005302 (PMC5289618; doi:10.1371/journal.pntd.0005302)
Supplement: S6 Table — (DOCX) [file pntd.0005302.s015.docx]

**Table S6:** Binding parameters of the productive poses of permethrin, deltamethrin, DDT and bendiocarb in the active sites of various *Ae. aegypti* CYP9J27 models.

| **Allele** | **Binding energy (Kcal/mol)** | | | |
| --- | --- | --- | --- | --- |
|  | **Permethrin** | **Deltamethrin** | **DDT** | **Bendiocarb** |
| **KL** | -10.1 | -10.3 | -8.6 | -7.5 |
| **JB** | -9.9 | -9.6 | -8.5 | -7.3 (2^nd^ Rank) |
| **KB** | -9.9 | -9.8 | -8.9 | -7.1 |
| **PG** | -9.6 | -9.6 | -8.8 | -7.2 (3^rd^ Rank) |
| **NO** | -9.1 | -9.3 | -5.3 | -5.1 |
